# Guanine Holes Are Prominent Targets for Mutation in Cancer and Inherited Disease

**DOI:** 10.1371/journal.pgen.1003816

**Published:** 2013-09-26

**Authors:** Albino Bacolla, Nuri A. Temiz, Ming Yi, Joseph Ivanic, Regina Z. Cer, Duncan E. Donohue, Edward V. Ball, Uma S. Mudunuri, Guliang Wang, Aklank Jain, Natalia Volfovsky, Brian T. Luke, Robert M. Stephens, David N. Cooper, Jack R. Collins, Karen M. Vasquez

**Affiliations:** 1 Division of Pharmacology and Toxicology, The University of Texas at Austin, Dell Pediatric Research Institute, Austin, Texas, United States of America; 2 Advanced Biomedical Computing Center, SAIC-Frederick, Inc., Frederick National Laboratory for Cancer Research, Frederick, Maryland, United States of America; 3 Institute of Medical Genetics, School of Medicine, Cardiff University, Cardiff, United Kingdom; University of Washington, United States of America

## Abstract

Single base substitutions constitute the most frequent type of human gene mutation and are a leading cause of cancer and inherited disease. These alterations occur non-randomly in DNA, being strongly influenced by the local nucleotide sequence context. However, the molecular mechanisms underlying such sequence context-dependent mutagenesis are not fully understood. Using bioinformatics, computational and molecular modeling analyses, we have determined the frequencies of mutation at G•C bp in the context of all 64 5′-NGNN-3′ motifs that contain the mutation at the second position. Twenty-four datasets were employed, comprising >530,000 somatic single base substitutions from 21 cancer genomes, >77,000 germline single-base substitutions causing or associated with human inherited disease and 16.7 million benign germline single-nucleotide variants. In several cancer types, the number of mutated motifs correlated both with the free energies of base stacking and the energies required for abstracting an electron from the target guanines (ionization potentials). Similar correlations were also evident for the pathological missense and nonsense germline mutations, but only when the target guanines were located on the non-transcribed DNA strand. Likewise, pathogenic splicing mutations predominantly affected positions in which a purine was located on the non-transcribed DNA strand. Novel candidate driver mutations and tissue-specific mutational patterns were also identified in the cancer datasets. We conclude that electron transfer reactions within the DNA molecule contribute to sequence context-dependent mutagenesis, involving both somatic driver and passenger mutations in cancer, as well as germline alterations causing or associated with inherited disease.

## Author Summary

A large number of DNA mutations identified in cells from patients with cancer or human inherited disease were analyzed to address a fundamental issue in human pathology, *viz*, the mutational mechanisms that cause irreversible changes to DNA. By using bioinformatics and computational methods, we found that mutations do not occur randomly, but instead affect specific bases, most often guanines flanked by other guanines or adenines. We attribute this effect to electron transfer, a chemical reaction known to underlie basic biological processes such as cellular respiration and photosynthesis. Certain types of carcinogens, oxidants or radiation can interact with DNA and abstract an electron. Our results imply that the ensuing sites of electron loss can migrate from their original position in the DNA to neighboring guanines where they become trapped, leading to further chemical modifications that may eventually result in mutations. Many of the mutations known to be important for tumor growth (driver mutations), as well as passenger mutations and mutations associated with inherited disease, appear to be caused by electron transfer. Beyond pathological mutations, electron transfer may represent a universal mechanism by which genetic changes occur in all life forms to drive population fitness over evolutionary time.

## Introduction

At least fifteen cancer genome sequencing projects were reported between 2007 and 2011 [1]–[15], and this number is now increasing very rapidly. These studies have been critical for addressing mechanisms of somatic mutation, such as those associated with single base substitutions (SBSs), which not only represent the vast majority of lesions in most patients, but also (in the case of some driver mutations) alter gene function, thereby initiating tumor development. Such investigations have demonstrated that SBSs do not occur randomly throughout the genome. Indeed, frequent C→T transitions have been noted at CpG dinucleotides [4], [9], [16]–[18], which are attributable to the high rate of spontaneous 5-methylcytosine (^5m^C) deamination at methylated ^5m^CpG sites [19], [20]. In individuals with a history of exposure to cigarette smoke or radio/chemotherapy, high proportions of G→T transversions, G→A and G→C substitutions at GpA and CpG dinucleotides, and A→T and A→G substitutions at TpA dinucleotides have also been reported [6], [8], [9], [21], suggestive of DNA damage through exogenous mechanisms [22], [23]. Likewise, large numbers of C→T transitions at YpC (Y = C/T) dinucleotides in melanoma in sun-exposed areas of the skin [11], [21], [24] have been attributed to cyclobutane pyrimidine dimer (CPD) formation following UV photoexcitation [25]. For less common types of substitution, such as T→C at ApT dinucleotides in hepatocellular carcinoma [16], underlying mutational mechanisms have yet to be proposed.

Studies aimed at identifying the mechanisms underlying the sequence context dependency of SBSs observed in inherited disease [26], cancer and phylogenetic analyses [20], [27]–[31] are few in number. A recent analysis of breast cancer genomes identified five types of trinucleotide motif enriched in SBSs, all of which contained either a CpG or a GpA motif [18]. Substitutions at CpG were attributed to ^5m^CpG deamination, whereas mutations at the GpA motif, which displayed sporadic clustering, were linked to enzymatic deamination of C at TpC by TC-specific cytosine deaminases [18]. Cluster analyses in other types of cancer led Roberts et al. to propose a similar mechanism for mutations at GpA sequences [32]. Indeed, recent work has identified APOBEC3B as a likely enzymatic source of C→T transitions in breast cancer [33]. In melanoma, Krauthammer et al. reported an enrichment of mutations at C in the context of 5′-TTTCGT-3′ motifs, a finding which was attributed to energy transfer along the pyrimidine-rich strand upon UV exposure [34]. Thus, although the influence of flanking bases on SBSs appears to extend beyond dinucleotide units, substantial gaps remain in our understanding of the mutational mechanisms involved. Elucidating these mechanisms is crucial, not only because they provide critical information on the earliest steps of cancer-associated mutagenesis, but also because they may account for inter-individual genetic variation as well as somatic age-related changes within the same individual.

Herein, we analyzed the frequencies of mutation at G•C bp in the context of all possible 4-bp 5′-NGNN-3′ units from >530,000 SBSs representing 21 cancer genomes, >77,000 germline mutations causing or associated with human inherited disease and 16.7 million benign germline single nucleotide variants (SNVs). The 64 combinations of 5′-NGNN-3′ motifs provided a suitable set size that was not too large to hamper sequence representation while doubling the length of base interactions relative to the commonly employed dinucleotide sequences [35]. In several cancer mutation datasets, but also in the germline mutations, the frequencies of substitutions correlated with the free energy of base stacking along the G-containing strand, as well as with ionization potentials, *i.e.* the energy required for abstracting an electron from the target guanines. Such behavior is consistent with an electron transfer mechanism as a consequence of one-electron oxidation reactions between the DNA molecule and radical species in the cell [22], [36], [37]. We conclude that electron transfer contributes to sequence context-dependent SBSs, not only in the context of cancer genomes but also in pathogenic germline mutations.

## Results

### SBSs Occur Preferentially at G•C Base-Pairs (bp)

**Figure 1:**
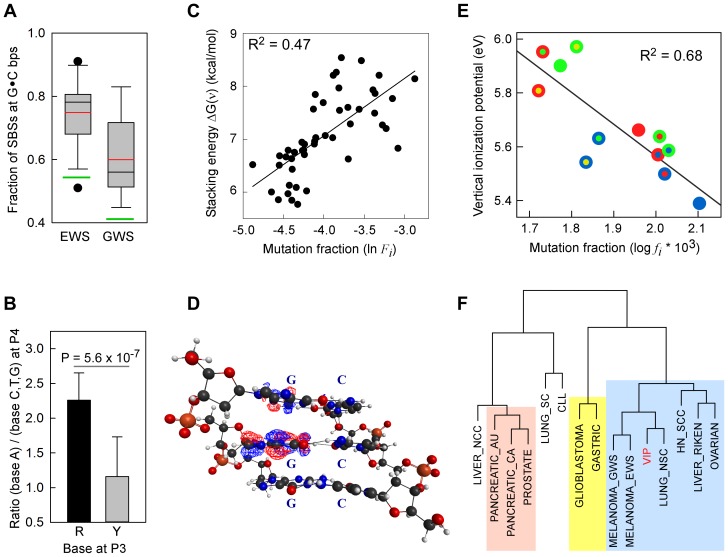
SBSs and VIPs. *Panel A*, whisker plot of the fractions of SBSs at G•C bp for the EWS and GWS datasets computed using AgilentV2 and Duke35 mappability counts, respectively; *red line*, mean; *black line*, median; *green lines*, average GC-contents in the mappable AgilentV2 (EWS) and Duke35 (GWS) sets. *Panel B*, NGRA sequences are enriched in SBSs in melanoma. *y-axis*, for each 4-member sequence combination with matching P1–P3 bases, the fraction of mutations at P4-A was divided by the average fraction of mutations at P4-(C/T/G); *x-axis*, P3 base composition; *R*, purine; *Y*, pyrimidine; mean ± SD; P-value from two-tailed *t*-test. *Panel C*, the *ln* of normalized fractions of mutated DGNN (D = A/G/T) sequences, *F_i_*, for the seven cancer datasets with −logP ≥3 for DGRN>DGYN (Table 1) were combined and plotted as a function of the average absolute free energy of base stacking, ΔG(ν), for each of the 48 DGNN sequences. *Panel D*, three-dimensional model of the (5′-GGG-3′)•(5′-CCC-3′) trinucleotide showing the LUBMO (lowest unoccupied beta molecular orbital) of the ionized sequence. *Panel E*, plot of the normalized fractions (*log f_i_*×10^3^) of mutated DGN sequences (Duke35 counts) for the Lung_nsc cancer dataset *vs.* VIPs; *outer circle*, 5′D base; *inner circle*, 3′N base; *blue*, adenine; *green*, guanine; *red*, thymine; *yellow*, cytosine. *Panel F*, agglomerative hierarchical clustering of 14 cancer genome datasets obtained from linear correlations with *ln* VIP values, as obtained from T_hg19 counts; *colored boxes*, elements found to be clustered at the 90% confidence interval.

We collected the publicly available data from cancer genome studies reported in PubMed between 2007 and 2011, together with the 5 largest datasets from the International Cancer Genome Consortium (ICGC) (Table 1). Twenty-one datasets, 13 from exome-wide (EWS) and 8 from genome-wide (GWS) sequencing scans, together represented cancers from 14 different tissues comprising 1,149 patient samples (1 to 393 samples per dataset) and 2 cell lines, for a total of 533,482 SBSs (Table S1). Additional SBSs from 3 other datasets were included in the analysis: a subset of nonsense and missense germline mutations of pathological significance in the context of inherited disease derived from the Human Gene Mutation Database (HGMD); a subset of splicing mutations from HGMD causing human inherited disease; and the set of SNVs from the “1000 Genomes Project” included in dbSNP build 129 (“rs” set), to allow direct comparison of the cancer-associated somatic mutations with germline mutations and polymorphisms present in the general population. The median fractions of SBSs that occurred at G•C bp were 0.78 (mean ± SD, 0.75±0.11) for the EWS datasets and 0.56 (mean ± SD, 0.60±0.13) for the GWS datasets (Fig. 1A), significantly higher than the average GC-content exome-wide (0.55) [38] and genome-wide (0.41), respectively [39] (both P values were ∼2.2×10^−16^, the smallest computable number by implementation of the binomial exact test in R). Thus, SBSs occurred more frequently at G•C bp, as compared to A•T bp, than expected by chance alone, both in cancer genomes and in the germline as a cause of inherited disease.

**Table 1:** List of datasets and SBSs at G•C base pairs.

*Dataset*	*Exome or genome wide seq.*	*Number of mutations*	*Fraction of SBS* 1 *at G•C bp*	*− logP CGNN vs. DGNN*	*− logP DGRN vs. DGYN*	*Number of samples*	*Reference*
Acute myeloid leukemia	EWS	1834	0.51	12	ns	1	[1]
Breast	EWS	1344	0.80	8	3	42	[2]
Chronic lymphocytic leukemia	GWS	4048	0.52	19	ns	4	[3]
Colorectal_1	EWS	1153	0.81	>3	ns	38	[2]
Colorectal_2	EWS	686	0.79	>3	ns	36	ICGC^2^
Gastric	EWS	4703	0.66	24	ns	22	[4]
Glioblastoma	EWS	2245	0.91	9	ns	27	[5]
Head_neck SCC (2 datasets)	EWS	9974	0.80	15	4	91	[6], [7]
Liver_ncc	GWS	142,791	0.50	23	ns	11	ICGC^3^
Liver_riken	GWS	175,094	0.45	20	5	16	ICGC^4^
Lung_nsc	GWS	50,666	0.78	10	9	1	[8]
Lung_sc	GWS	22,905	0.65	22	2	1	[9]
Melanoma_ews	EWS	2809	0.88	2	4	14	[10]
Melanoma_gws	GWS	33,339	0.83	ns	6	1	[11]
Multiple myeloma	EWS	1204	0.68	16	ns	38	[12]
Ovarian carcinoma	EWS	18,121	0.73	22	ns	316	[13]
Pancreatic_au	EWS	1864	0.70	14	ns	87	ICGC^5^
Pancreatic_ca	GWS	28,041	0.53	24	ns	5	ICGC^6^
Prostate	GWS	28,189	0.56	23	ns	7	[14]
Mixed	EWS	2472	0.78	13	3	393	[15]
Inherited missense/nonsense mutations	na	67,513	0.68	26	ns		BIOBASE^7^
Inherited splicing mutations	na	9907	0.65	2	2		BIOBASE^7^
1000 Genomes Project	GWS	16,744,224	0.56	38	3^9^		1000 GP^8^

Mutation datasets were derived from sixteen cancer genome studies published between 2007 and 2011, five cancer genome studies reported by the ICGC, two datasets of germline pathogenic mutations from HGMD and the “rs” entries (*i.e.* entries in the dbSNP membership, build 129) from the 1000 Genomes Project. −logP values were obtained from two-tailed *t*-tests (*z*-tests for the colorectal cancer datasets) using the NGNN counts from AgilentV2 (EWS) and Duke35 (GWS).

1 SBS, single base-pair substitutions;

2 http://dcc.icgc.org/ (JHU, US);

3 http://dcc.icgc.org/ (RIKEN, JP);

4 http://dcc.icgc.org/ (NCC, JP);

5 http://dcc.icgc.org/ (QCMG, AU);

6 http://dcc.icgc.org/ (OICR, CA);

7 http://www.biobase-international.com/product/hgmd (HGMD Professional Human Gene Mutation Database, release 2011.3);

8 http://www.1000genomes.org/ (2011 release).

### SBS Frequencies Are Modulated by Flanking Base Composition

We addressed the sequence-dependent occurrence of SBSs at G•C bp by retrieving the 5′-NGNN-3′ and their complementary 5′-NNCN-3′ sequences (henceforth referred to as NGNN), either genome-wide or exome-wide, and calculating the fractions of mutated motifs, *f*(NGNN), for each of the 64 sequence combinations. There were two confounding factors in computing *f*(NGNN): the first related to the fact that only certain portions of the human genome may be effectively mapped by next-generation sequencing; the second originating from the various methodologies used during base-variant mapping and calling (see Materials and Methods). Therefore, we first assessed the relative representation of NGNN sequences in the homologous portions (Segmental Duplications, repetitive elements and simple repeats) as compared with the unique portions of the human genome (Text S1 and Table S1). These analyses indicated that CGNN motifs are significantly overrepresented in Segmental Duplications (Text S1 and Table S2). We then used the genomic mutation sites (Table S3) to compute *f*(NGNN) according to three methods (Duke35, CRG50 and T_hg19) for the GWS datasets and three methods (AgilentV2, CGR50_exons and T_exons) for the EWS datasets (Text S1), and used these fractions to assess the extent to which base composition at positions 1, 3 and 4 (P1, P3 and P4) would influence G•C mutations at position 2 (P2) for different classes of NGNN sequences. Mutations were observed at each NGNN sequence combination with the exception of the two small colorectal cancer datasets (Table 1; Table S4, Panel *A*). Thus, for these two datasets, *z*-tests, rather than *t*-tests, were used to assess statistical significance. Irrespective of the mapping method used, *f*(CGNN) mean values were significantly greater than *f*(DGNN) (D = A/G/T) mean values in all cancer (2–11 fold, depending on cancer type with gastric and colorectal cancers displaying the largest differences) and germline mutation datasets (Table S4, Panels *B*–*D*), with −logP values ranging from 8 (Breast; 9.3±3.3×10^−5^ for CGNN *vs.* 4.0±2.6×10^−5^ for DGNN, AgilentV2 counts) to 41 (1000GP; 6.9±1.3×10^−2^ for CGNN *vs.* 7.5±1.5×10^−3^ for DGNN, CRG50 counts) (Table 1). Exceptions were the two Melanoma datasets, for which such differences were modest due to considerable variability in the data (2.2±3.4×10^−4^ for CGNN *vs.* 0.9±1.2×10^−4^ for DGNN, P-value 0.02 for Melanoma_ews, AgilentV2 counts; 4.9±7.5×10^−5^ for CGNN *vs.* 2.4±2.8×10^−5^ for DGNN, P-value 0.06 for Melanoma_gws, Duke35 counts). Thus, with the notable exception of the melanomas, the CpG dinucleotide, a substrate for cytosine methylation, represents a strong mutation hotspot, both in the soma and the germline.

For the DGNN sequences, a P3-purine significantly increased the proportions of P2 SBSs, *f*(DGRN), compared to a pyrimidine, *f*(DGYN) in 7 cancer types (−logP values 3–9), including lung, head and neck, and melanoma (Table 1), for which associations with exposure to either cigarette smoke or sunlight have been documented. An additional dataset, Lung_sc, from the established cell-line NCI-H209 displayed modest *f*(DGRN)>*f*(DGYN) (P-value ∼0.04). Again, in no cases were P-values contradictory based upon the mapping method used (Table S4, Panels *B*–*D*). The data for the melanomas were particularly striking since P3-A increased the fractions of mutation at P2-G by ∼10-fold relative to DGBN (B = C/G/T) (14.3 *vs*. 1.7×10^−5^ for Melanoma_gws, P-value 9.1×10^−4^; and 68.4 *vs*. 6.4×10^−5^ for Melanoma_ews, P-value 2.4×10^−5^; according to Duke35 and AgilentV2, respectively, Table S5). In addition, the CGAN motifs displayed ∼3-fold higher mutation fractions than the DGAN motifs (14.3 *vs*. 4.9×10^−5^, P-value 0.019; and 68.4 *vs*. 20.5×10^−5^, P-value 2.8×10^−3^) in Melanoma_gws and Melanoma_ews, respectively, although a mutagenic role for CpG methylation was not apparent (*i.e.* the CGBN and DGBN fractions were indistinguishable, P-values ∼0.6–0.7). In addition, for the two melanoma datasets, P4-A significantly increased (2.2±0.2 fold) mutation at P2-G (Fig. 1B) when P2 and P4 were separated by a purine (P-value 5.6×10^−7^). Additional analyses in four melanoma datasets [17], [21], [34], [40] confirmed this finding (ratio for the eight possible NGRA/NGRB groups in these four datasets was 2.2±0.5 and 2.2±0.3 for the combined six datasets, Table S6). The increase in mutation at P2-G, relative to P4-C and P4-T, was also observed for P4-G; however, this effect was less consistent than P4-A and was observed more frequently when P3 was occupied by a guanine (18/23 cases) rather than an adenine (5/23 cases), Table S6.

In summary, SBSs at P2-G were dependent upon the sequence composition of the 3′-nearest neighbor in a number of different cancer types; in melanoma, this effect extended to the next 3′ base when bridged by a purine. Thus, GpR and GpRpA sequences constitute mutational hotspots that render the 5′ G sensitive to mutation. Further analyses performed in individual cancer samples (Text S1, Figure S1 and Table S4, Panels *B–E*) indicated that biological mechanisms, rather than differences in variant-calling algorithms or variability between individual samples, were the likely causes of such mutational patterns.

### Electron Transfer and Sequence-Dependent SBSs

Guanine is the most readily oxidized base [41] and its ionization energy, *i.e*. the energy required to abstract an electron, depends upon the identity of the flanking nucleotides [42]–[45]. Substantial work performed with model DNA sequences *in vitro* has shown that, following one-electron oxidation reactions, the sites of electron loss (hole) migrate efficiently (rate constants ∼10^7^ s^−1^) from the original locations to distant sites, where they become trapped in troughs of low ionization energy, most often at GG and GGG sequences [36], [44], [46]–[48]. Because oxidative DNA damage occurs spontaneously in the cell, we tested whether the sequence-dependent SBS patterns were consistent with a mutagenesis model that included: a) loss of an electron within the NGNN sequences; b) hole migration to the P2-guanine; and c) chemical modification of the P2-guanines leading to base substitutions [22].

### Absolute Free Energy of Base Stacking

The binding energy of single-stranded stacked bases is presumed to be dependent upon the affinity of interactions, or the extent of electron sharing, between π orbitals across bases [49]. The free energy of base stacking, rather than hydrogen bonding, has been reported to be the major source of stability in duplex DNA [50]. Hence, we expected that strongly interacting bases would be more prone to one-electron oxidation and, hence, to higher SBS rates than weakly interacting bases. To this end, we used the absolute free-energy values of base stacking between non-bonded bases, ΔG(ν), derived from a theoretical study [51] using a continuum solvation model and Amber force field to assess the relationships with *f*(DGNN) values, as we previously employed [52]. For 5/7 datasets with *f*(DGRN)>*f*(DGYN) (Table 1), *i.e.* two melanomas, Lung_nsc, Liver_riken and Mixed, a significant positive correlation existed between the fraction of mutated DGNN sequences and free energies of base stacking (Table S7, Panel *A*; *r* ^2^ 0.10–0.71; P-values <0.001–0.031). The normalized mutation fractions for the combined 7 datasets also displayed significant correlation (*r* ^2^ 0.47; P<0.001; P(α)_0.05_ = 1.000; Fig. 1*C* and Table S7, Panel *A*; *f*(DGNN) were according to Duke35 and AgilentV2 mappability).

### Vertical Ionization Potentials (VIPs)

VIP, the minimum energy required to abstract an electron, is commonly used as a measure of one-electron oxidation reactivity [41]. We modeled the susceptibilities of G-centered DGN double-stranded trimers to oxidation *via* quantum chemical computations of VIPs. These analyses are expected to compare favorably with data obtained from the computationally more demanding tetramers; the VIPs for tetramers would be expected to be lower than for trimers while maintaining similar sequence-dependent rankings for P2 [53]–[56]. The VIP values for the 12 trimers were ∼30–40% lower than that of an isolated guanine (Table 2) whose VIP estimate was close to the experimentally determined lowest band maximun [57]. The trimer with the lowest VIP was GGG, in agreement with prior calculations [42]–[45] and all trimers containing a GG doublet had lower VIPs than those with a single G. In addition, a purine at the 3′ position was consistently associated with lower VIPs than a pyrimidine at the 3′ position. Thus, DNA sequence context affects VIPs, in accordance with guanine reactivities to oxidative reactions *in vitro* [42], [44].

**Table 2:** Vertical ionization potentials (VIPs) of guanine-centered DGN sequences.

Sequence	Vertical Ionization Potential (eV)
G*G*G	5.39
G*G*A	5.50
G*G*T	5.54
G*G*C	5.63
A*G*G	5.57
T*G*G	5.59
T*G*A	5.64
A*G*A	5.66
A*G*T	5.81
T*G*T	5.90
A*G*C	5.95
T*G*C	5.97
*G*	8.02^a^
*G* (exp)	8.26^b^

VIPs for the centrally (italicized) guanine computed at the M06-2X/6-31G(*d*) level of theory;

a VIP of free unalkylated guanine;

b from reference [57].

Inspection of the lowest unoccupied beta molecular orbital (LUBMO) for each DNA trimer cation, [DGN]^+^, in which the ionization state was modeled by removing an electron, showed that the electron hole invariably had π character with high densities at the central guanine (Fig. 1*D*), or at the 5′G in the GGH (H = A, C, T) sequences, consistent with previous work [42], [55], [56], [58], [59], implying that P2G was a frequent site for one-electron oxidation reactions. Analyses between *f*(DGN) and VIP values displayed significant correlations for 4/5 datasets that also revealed a correlation with ΔG(ν), *i.e.* melanomas, Lung_nsc and Liver_riken (as per Duke35 and AgilentV2 mappability; Figure 1*E*, Figure S2 and Table S7, Panel *B*; *r* ^2^ 0.54–0.75; P-values<0.001–0.007). Notably, robust correlation was also evident when the *f*(DGN) data from all 18 cancer datasets were normalized and then computed as average values (Table S7, Panel *B* and Figure S2, Panel *D*, *r* ^2^ 0.40; P-value 0.026; P(α)_0.05_ 0.615). The regression coefficients obtained using the T_hg19 and T_exons mappability data for the datasets with >2,000 SBSs were also used to perform hierarchical clustering based on absolute Manhattan distances (Fig. 1*F*). At a >90% confidence interval, this yielded three clusters, the largest of which contained the same cancer datasets, with the exception of Ovarian carcinoma, that also displayed *f*(DGRN)>*f*(DGYN) ratios (Table 1). In summary, both base stacking and VIP data support the conclusion that electron transfer in DNA represents a significant mechanism for sequence context-dependent mutagenesis in cancer.

### Sequence-Dependent SBSs in Cancer-Associated Genes

**Figure 2:**
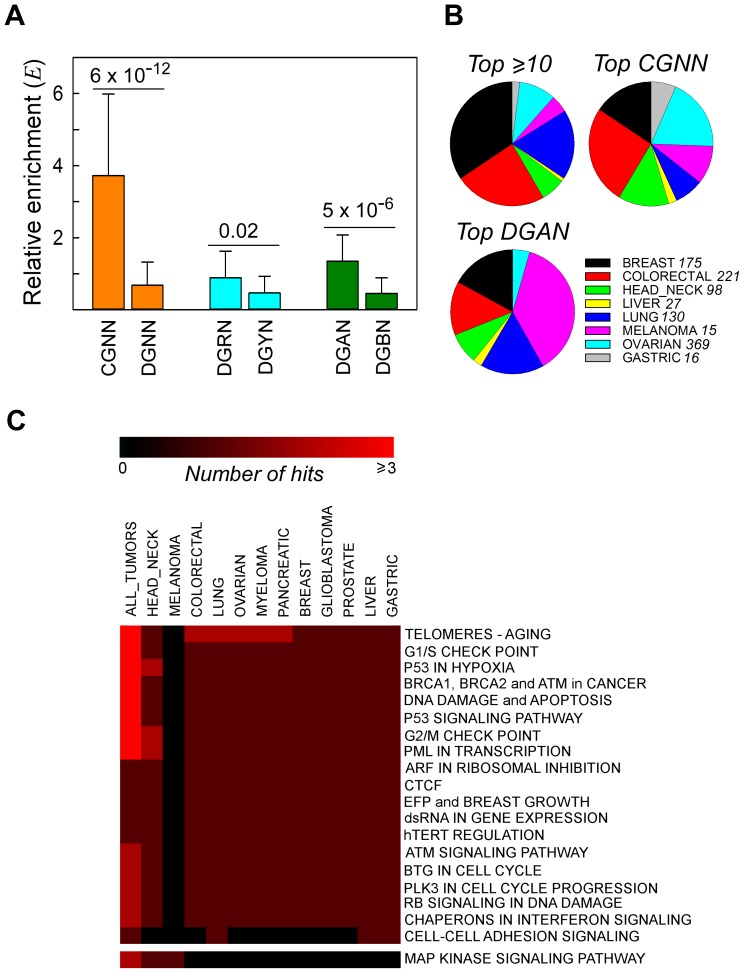
Recurrent NS substitutions display sequence context-dependent patterns of mutation. *Panel A*, relative enrichment, *E_i_*, of NGNN sequences carrying NS substitutions that recurred in different patient samples in all cancer types. *CGNN*, the 16 motifs beginning with a CpG step; *DGNN*, the 48 motifs not beginning with a CpG step; *DGRN*, the 24 motifs with a purine following the mutated G; *DGYN*, the 24 motifs with a pyrimidine following the mutated G; *DGAN*, the 12 motifs with an adenine following the mutated G; *DGBN*; the 36 motifs without an adenine following the mutated G; mean ± SD. *Panel B*, distribution of the top recurrently mutated genomic positions across the most abundant cancer types; *top ≥10*, A•T- and G•C-containing hg19 coordinates flanked by any sequence and mutated at least ten times (range 10–40 times, 16 coordinates); *top CGNN*, the three most commonly mutated CGNN sequences (CGTC, *E_i_* = 9.55, 13 coordinates; CGGA, *E_i_* = 5.99, 10 coordinates; and CGTG, *E_i_* = 5.66, 10 coordinates); *top DGAN*, the three most commonly mutated DGAN sequences (TGAT, *E_i_* = 2.79, 10 coordinates; GGAA, *E_i_* = 2.04, 10 coordinates; TGAA, *E_i_* = 1.97, 10 coordinates), see also Table S9. For each cancer type with at least 1000 NS substitutions, the fraction *S* was plotted on the pie charts. *Panel C*, pathway-level heatmap with the clustering of commonly hit pathways containing genes with recurrent NS substitutions amongst all tumor types. *Number of hits*, number of genes belonging to the same pathway or network.

Driver mutations include non-synonymous (NS) substitutions that play a key role in cancer initiation and progression. To assess whether *bona fide* driver mutations also occurred in a sequence context-dependent manner, we examined the NS substitutions that altered the same genomic coordinate in more than one patient sample, and the genes affected (Text S1, Figure S3 and Table S8). For the 224 recurrent NS substitutions at G•C bps, we calculated the relative enrichment *E* for each of the 64 NGNN motifs, a value which is expected to approximate to 1 if the base substitutions are completely independent of flanking sequence. *E* values were greater for the CGNN than for the DGNN (D = A/G/T) sequences (Figure 2, Panel *A*). Among the DGNN sequences, P3-purines were associated with significantly more mutations than P3-pyrimidines, a difference that was attributable to the presence of P3-A (DGAN>DGBN, B = C/G/T). This trend remained unaffected after the 45 entries from the two melanoma datasets [which showed *f*(DGAN)>*f*(DGBN) in the respective EWS and GWS screens (Figure 1, Panel *B*)] were removed (*E*(DGAN) = 0.92±0.52; *E*(DGBN) = 0.39±0.43; P = 0.001). Thus, recurrent NS substitutions occurred preferentially at CpG and GpA dinucleotides in cancer genomes, mirroring the sequence context-dependent pattern of SBSs observed both genome-wide and exome-wide (Figure 1 and Table 1). Of the 18 codon changes that recurred >12 times, 7/10 affected NGNN sequences and 5/10 occurred at CGNN sequences, all in well-established cancer genes (Table S9, Panel *A*). Likewise, the most commonly mutated CGNN (Table S9, Panel *B*) and DGAN (Table S9, Panel *C*) motifs affected known driver mutations, alongside several novel candidate genes and driver mutations (Table S9), including p53^R248G^, which has been reported to alter protein function (http://www-p53.iarc.fr), and *GRHL3*, *WNK3*, *EPHB1*, *ADCY2*, *GSK3B* and *LRRN3*, which are not currently listed in the cancer gene census (http://www.sanger.ac.uk/genetics/CGP/Census).

### Tissue Distribution and Networks Affected

To examine whether recurrent NS substitutions occurred equally in all tumor tissue types, we determined the relative distributions of the most frequently mutated genomic coordinates after normalizing for both tissue representation and the total number of SBSs per dataset; in the absence of any bias, each tissue would contribute 12.5%. The four genes with ≥12 recurrently mutated genomic coordinates (*TP53*, *KRAS*, *PIK3CA* and *BRAF*) (Table S9, Panel *A*) were predominantly of breast (34%), intestine (24%) and lung (18%) origin (Figure 2, Panel *B*). The three most commonly mutated CGNN sequences (CGTC, CGGA and CGTG; *S* = 9.5, 6.0 and 5.7, respectively) were found in genes mutationally altered in the intestine (26%), ovary (19%) and breast (15%), whereas the most commonly mutated DGAN sequences (TGAT, GGAA and TGAA; *S* = 2.8, 2.0 and 2.0, respectively) were found predominantly in genes altered in melanoma (37%), breast and lung (17% each) (Table S9). By contrast, these mutated motifs were underrepresented in the liver (≤2%). Of the 64 codons affected, 6 (3 in *TP53*, 2 in *PIK3CA* and 1 in *GNAS*) are known driver mutations, 4 introduced stop codons into *TP53*, and 26 occurred within genes whose involvement in cancer is strongly suspected (Table S9 and http://www.sanger.ac.uk/genetics/CGP/Census). Thus, although high-confidence driver mutations occurred preferentially at CGNN and DGAN motifs, their occurrence between tissues was highly asymmetrical, with DGAN mutations occurring predominantly in tumors of the skin.

Finally, we used pathway analyses to survey the 150 recurrently mutated genes (Figure 2, Panel *C*). In all tumor tissues, 18 pathways/networks related to cell-cycle checkpoints and the DNA damage response were found to be compromised in all types of tumor, the sole exception being melanomas in which only the MAP kinase signaling pathway was consistently altered. A similar pattern was revealed when all NS substitutions were analyzed, irrespective of whether the data from all patients were merged (Figure S4, Panel *A*) or plotted separately (Figure S4, Panels *B* and *C*). The highest-ranking pathways were dominated by *TP53* mutations in most tumor types (Figure S4, Panels *B* and *C*), with the exception of pancreatic cancers in which *KRAS* mutations dominated. Both p53 and KRAS proteins are known to act on parallel signaling cascades that regulate TERT, the active reverse transcriptase component of telomerase that controls the stability of chromosome ends (http://www.biocarta.com/pathfiles/h_telPathway.asp). Hence, although critical pathways represent common targets for oncogenic transformation, the altered genes may vary between different patients or organ/tissue types. In summary, a distinction emerged between melanoma and the other types of cancer, both with regard to the sequence contexts targeted by driver mutations, DGAN *vs.* CGNN sequences, and to the pathways that hosted these mutations, MAP kinase *vs.* p53-associated signaling pathways.

### Guanine Is Preferentially Targeted by Pathogenic Germline Mutations

**Figure 3:**
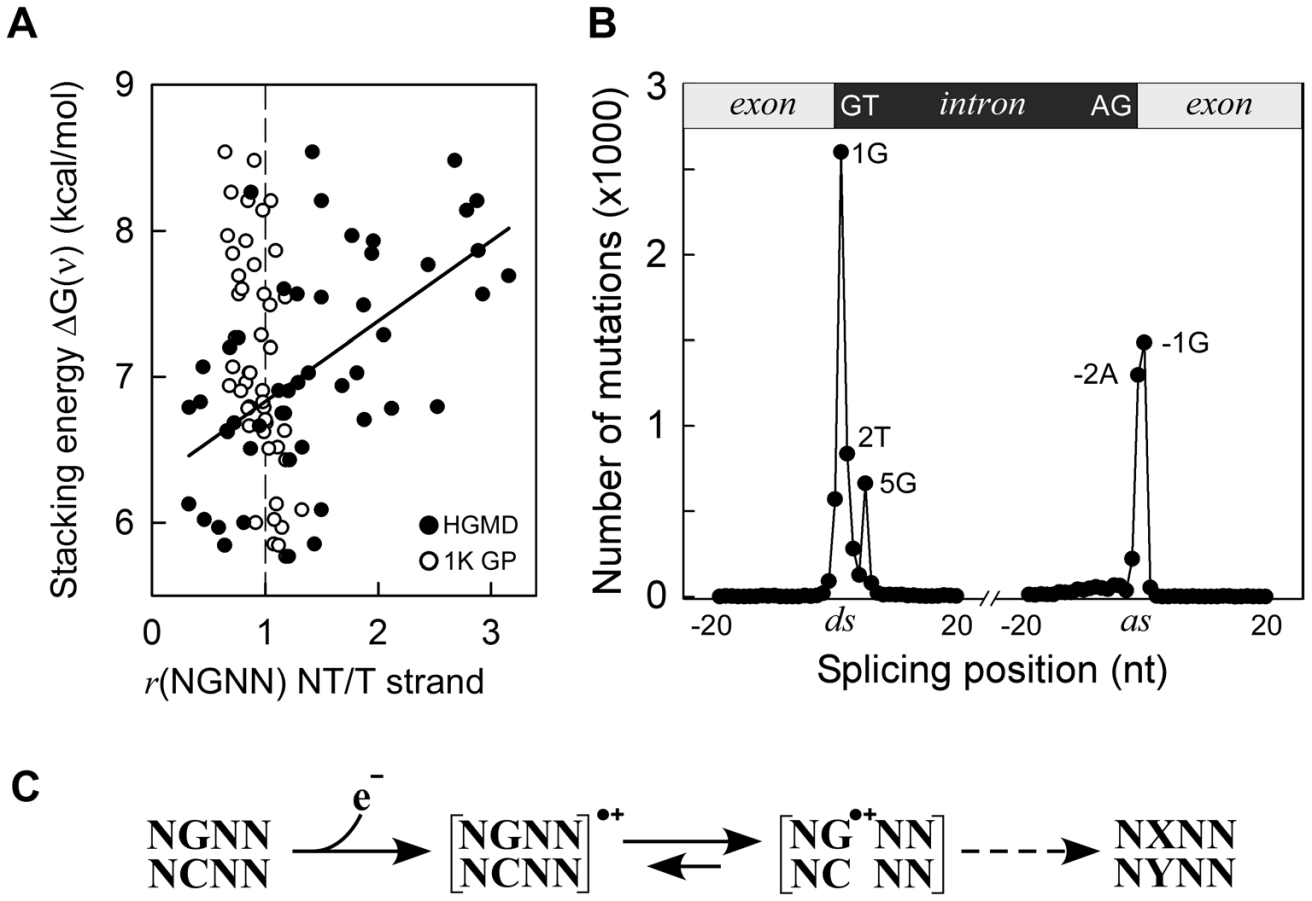
Germline mutations are affected by transcription. *Panel A*, HGMD dataset; *y-axis*, as in Figure 1C; *x-axis*, ratio of mutated NGNN sequences in protein coding genes containing the P2-guanine base on the non-transcribed (*NT*) *vs*. transcribed (*T*) strand; *solid circles*, HGMD dataset (*r* ^2^ 0.32, P(α)_0.05_ 0.991, P<0.001); *open circles*, 1000 Genomes Project dataset. *Panel B*, inherited splicing mutations dataset; *top*, cartoon of exon-intron boundaries showing the conserved GT and AG bases at the donor (*ds*) and acceptor (*as*) splice sites; *bottom*, graph of splicing mutations; *y-axis*, number of SBSs; *x-axis*, position of SBSs relative to +/−20 nt of splice junctions; *Panel C*, model for sequence-dependent SBSs in cancer and human inherited disease. In the first step, an electron is lost from within a tetranucleotide sequence, leaving a hole. In the second step, the hole migrates to and from various competing sites, including nearby bases and chromatin-associated amino acids (not shown), eventually being trapped by a guanine base. The resulting guanine radical cation either causes DNA-protein crosslinking or undergoes subsequent chemical modifications. If the modified base is not corrected by DNA repair, it may give rise to a mutation (X-Y base-pair) as a result of error-prone DNA polymerase synthesis during DNA replication (dashed arrow).

In the HGMD missense/nonsense mutation dataset, approximately 68% of SBSs occurred at G•C bps, a proportion similar to the EWS cancer datasets, although correlations with ΔG(ν) or VIPs were absent (Table S7, Panel *B*) and no enrichment for DGRN sequences was apparent (Table 1). However, *r*(DGNN), which measured the fraction of mutated DGNN motifs relative to the direction of transcription, revealed that the P2 position was more likely to contain a guanine on the non-transcribed strand, relative to the transcribed strand, when stacking interactions with neighboring bases were high (*r* ^2^ 0.32; P-value<0.001; P(α)_0.05_ 0.991; Figure 3, Panel *A*). No such behavior was evident in the cancer datasets (not shown), whereas limited bias was observed in the 1000 Genomes Project dataset (Figure 3, Panel *A*). Thus, transcription led to a pattern of sequence context-dependent SBSs among pathogenic germline mutations, which mirrored that observed in several cancer genomes.

The HGMD dataset of inherited splicing mutations contained 9,907 SBSs that may be assumed to adversely affect RNA processing; 8,308 (84%) of these mapped to within 5 bases of donor and acceptor splice junctions (Figure 3, Panel *B*). Strikingly, although the canonical GT and AG intronic splice junctions at the donor AG∧**GT**AAGT and acceptor C**AG**∧GT sequences were found to be >99.9% conserved in the RefSeq dataset of human genes (Figure S5 *top*), only three positions, *i.e.* AG∧**G**TAAGT and C**AG**∧GT, were frequently mutated (1,297–2,601 SBSs, 65%), the T-containing donor position being only modestly affected (836 SBSs). We also used the 1000 Genomes Project dataset to assess the extent of splicing variation in the general population (Figure S5 *middle*); the smallest number of SNVs occurred at all 4 highly conserved positions, as expected. By contrast, in the cancer datasets, the number of SBSs around splice junctions was found to be independent of sequence conservation (Figure S5 *bottom*). In summary, pathological germline splicing mutations preferentially targeted those positions that exposed a purine base to the non-transcribed strand during DNA transcription.

## Discussion

Large-scale next-generation sequencing projects of cancer genomes are providing an unprecedented opportunity to address the key issue of the nature of the underlying mechanisms of base substitution in tumorigenesis. This issue is generally approached by analyzing the types of base substitution specific to the cancer tissue, *i.e.* mutation spectra, based on the assumption that different types of base substitution originate via different mutational mechanisms, as assessed by animal model systems [4], [6], [8], [9], [16]–[18], [21], [60]–[63]. We have chosen the alternative approach of addressing the sequence-context dependency of single base substitution, with the expectation of shedding light on the earliest step(s) in the process of mutagenesis, *i.e.* the susceptibility of DNA to base modification. Once modified, a base would then undergo various types of substitution based upon the type of modification it incurred, its interactions with DNA repair and replication systems and, possibly, the tissue of origin [62].

As revealed by correlations with VIPs and absolute free energies of base stacking, we uncovered a direct correlation between electronic coupling along the DNA chain, leading to electron transfer, and sequence-dependent SBSs, both in human cancers and as a cause of inherited disease. Thus, charge transfer appears to be the earliest event in the mutational mechanism acting along the path leading to base substitution in cancer. Electron transfer is the simplest chemical reaction and is known to underlie a number of fundamental biological processes such as cellular respiration and photosynthesis. By establishing the relevance of charge transfer to mutational changes in the DNA molecule, our study enables improved predictions of the relative contribution of individual mutagenic processes and DNA repair activities to cancer (Donohue *et al*., unpublished data). The somatic and germline settings studied here are qualitatively and quantitatively different and quite distinct from one another. In the former, very large numbers of somatic mutations occur as a consequence of the disease state whereas in the latter, only one or two germline mutations are generally involved in disease etiology. Despite these fundamental differences, similar sequence context-dependencies are evident, which are explicable in terms of the intrinsic physical properties of DNA, *i.e.*, free energy of co-axial base stacking and electronic coupling among flanking bases. We propose a model for SBSs that includes one-electron oxidation reactions (Figure 3, Panel *C*). In the first step, abstraction of an electron from DNA (base or sugar) by a radical species, either endogenous or exogenous, creates an electron hole. In the second step, the electron hole migrates reversibly to various competing sites, including flanking or more distant bases, as well as other molecules and contacting chromatin-associated amino acids, causing in some instances DNA-protein crosslinks [64]. Guanines with the lowest ionization potentials, as determined by neighboring bases, are the strongest hole-attracting sites. The resulting radical cations (G^•+^) are then expected to undergo a number of chemical modifications, leading to a variety of stably modified bases, including 8-oxoG, a key toxicological lesion, oxazolone, imidazolone and others, some of which can result in base changes during DNA replication if left unrepaired [22], [65]–[67]. Guanine-protein crosslinks may also lead to SBSs [68].

GpA, which we confirmed to be a key mutation hotspot [6], [8], [9], [21], [61], [63] and found to be enriched in sporadically clustered non-synonymous substitutions (Table S10), would therefore yield mutations through electron transfer [36], [37], [42], [69], tissue-specific deamination [33] and photoexcitation, leading to cyclobutane pyrimidine dimers (CPDs) in melanoma [10], [11]. We also identified NGRA, and to a lesser extent NGRG, sequences as mutation hotspots specific to melanoma. Attempts to determine whether mutations at NGRA might have been caused by UV-photosensitization or electron transfer, based on mutation spectra analyses (see Introduction), were uninformative since base substitution patterns were heavily sequence-context dependent. For example, in the context of these melanoma datasets, P2-G in the TGTT motifs underwent G→A:G→T substitutions in the relative proportions 17∶75, whereas for the TGCC motifs this ratio was 81∶13. Hence, sequence context determines the outcome of single base substitution in a manner that still eludes complete understanding. Nevertheless, if electron transfer reactions were involved, then P4-A would be expected to exert stronger effects than P4-G on mutations at P2-G, since hole trapping is much weaker on adenine than on guanine bases [47].

The CpG dinucleotide was found to be a consistent mutational hotspot, both in cancer and the germline, a result that generalizes the conclusions drawn from previous studies [4], [9], [16]–[18], [63]. The high frequency of C→T transitions at CpG dinucleotides is generally attributed to high rates of deamination of 5-methylcytosine resulting from methylation of CpG sites [9], [19], [20], [62]. However, other mechanisms have been proposed [60], [62], such as enhanced susceptibility of methylated CpG sites to damage by physical and chemical genotoxic agents [62]. This latter interpretation would be consistent with our finding that electronic coupling is an important factor in establishing the hierarchy for base modification in DNA. During the course of normalizing mutation fractions by genome mappability, we noted an enrichment of CGNN sequences in Segmental Duplications. Nakken et al. [70] reported a higher density of CpG islands in Segmental Duplications than in unique chromosomal regions, whereas Xie et al. found methylation-associated SNP clusters to be more prevalent in Segmental Duplications than in unique regions [71]. Thus, the prevalence of CGNN-associated SBSs may well be greater than our study indicates.

A confounding factor in our analyses is the relatively small number of SBSs, particularly in EWS datasets, which caused large variations in *f*_i_ values. Indeed, three of the four datasets that displayed high-confidence (*i.e.* P<0.05 and P(α)_0.05_>0.800) correlations between SBSs and ΔG(ν) or VIPs were obtained from genome-wide studies. Combining all *f*_i_ values into a single group (Figure S2, Panel *D*) only alleviates the problem, since the *f*_i_ values for each dataset are given the same weight. Nevertheless, the ensuing “cautiously significant correlation” is consistent with a role for electronic coupling in cancer-related mutagenesis. A second confounding factor is the multiple roles that the GpA dinucleotide plays in mutagenesis, as eluded to earlier. In the case of melanomas, if the numbers of mutated NGAN (and NGA) sequences were dominant, this might cause chance correlation with ΔG(ν) or VIP values, when in fact most mutations could arise from CPDs on the complementary strand. Correlations for both the Melanoma_gws and Melanoma_ews datasets remained highly significant (P<0.002; P(α)_0.05_ 0.920–1.000) when the *f*_i_ values for the NGAN (or NGA) sequences were excluded from the analyses, thereby confirming a role for charge transfer. This conclusion is further supported by the observation that electronic coupling and photo-induced energy transfer reactions at pyrimidine dimers occur simultaneously and impinge on one another [72]–[74].

In cancer, the subset of mutational changes resulting from NS substitutions that recurred in different patient samples displayed the same enrichment of mutations at CpG and GpA sequences as the exome-wide and genome-wide sequence alterations, supporting the notion of common underlying causes, *i.e.* cytosine methylation, electron transfer (this study), enzymatic cytosine deamination and CPD formation (in melanoma) [10], [11], [17]. These commonalities suggest that the mechanisms involved in generating “driver” tumor initiating mutations are likely to be similar to those involved in generating the bulk of subsequent “passenger” mutations. Hodis et al. [75] reached a similar conclusion using a quite different approach. Thus, electron transfer appears to be involved in both the early (driver mutations) and late (passenger mutations) phases of tumorigenesis, particularly in tissues of epithelial origin. Recurrent NS substitutions were observed predominantly in gene networks associated with p53 function in all tumor types, the exception being melanoma where a preponderance of mutations at GpA segregated with genes of the MAP kinase signaling pathway. The reason for this distinction remains unclear; however, the critical role played by the MAP kinase signaling pathway in melanocyte proliferation in response to UV damage [76] suggests that positive selection may have been a contributory factor.

The results of the HGMD data analysis support the occurrence of electron transfer in germline mutagenesis associated with human inherited disease, although sequence context-dependent mutagenesis was evident only when mutations were mapped onto the non-transcribed strands of genes. Guanines modified by oxidative DNA damage are repaired predominantly by base excision repair (BER) [77], [78]. Since oxidative DNA damage occurs more efficiently in single-stranded DNA than in double-stranded DNA [79], [80], oxidative guanine lesions may have formed more frequently on the single-stranded, non-transcribed, strand than on the DNA:RNA duplex during transcription. Thus, a greater number of lesions would be expected to escape BER on the non-transcribed strand than on the transcribed strand. In cancer cells, the large number of mutations that generally accumulate during tumor growth could have masked this bias. An alternative or additional possibility is that transcription-coupled nucleotide excision repair, a mechanism that processes bulky DNA adducts and which selectively corrects errors on the transcribed DNA strand [81], might have contributed to the strand asymmetric mutations observed in the HGMD dataset [9], [11], [12], [82], [83]. In similar vein, we interpret the selectivity of mutations at purines on the non-transcribed strands of splice junctions as a consequence of oxidative damage, whose effect could have been prolonged by the pausing of transcription-coupled splicing at splice junctions [84]. With the number of sequenced genomes rapidly increasing, it will be of great interest to ascertain whether electron transfer constitutes a general mutational mechanism that is common to all forms of life.

## Materials and Methods

### Datasets

We collected the publicly available data from cancer genome studies reported in PubMed from 2007 through December 2011 [1]–[15] together with the 5 largest datasets available from the International Cancer Genome Consortium (ICGC). The cancer genome datasets varied widely in terms of sequencing strategies, mapping techniques and variant-calling algorithms, implying that the power to detect SBSs may differ depending upon the datasets and methodologies used [85]. However, all studies excluded base variants present in matched-control tissues, such that the reported SBSs were changes attributed to somatic mutations in the tumor tissue. Matched controls were used for all patient samples. On average, between 6 [15] and 1834 [1] tumor-specific SBSs were reported in the EWS studies (between 1012 [3] and >50,000 [8] in the GWS studies) (Table 1), which is ∼1–3 orders of magnitude lower than the numbers of non-synonymous and splice-site variants noted on average in whole-exome studies [86]. In addition to normal-tumor matched samples, single nucleotide polymorphisms present in dbSNP databases or in the Venter and Watson genomes [1], [10], [11] were also used to exclude common base variants. Differences in variant-calling power were mitigated in our study since we examined relative proportions of mutated sequences, rather than absolute mutation fractions.

A second source of variation in detecting SBSs among the cancer genome studies was the sequencing instrument used. Illumina sequencers have been reported to yield systematic base-call errors, especially at the last base of context-specific GGC and GGT sequences, which affect either the forward or reverse strand, and at inverted repeats [87], [88]. The sequencing technologies employed included Illumina genome analyzers, SOLiD next-generation DNA sequencing, ion semiconductor sequencing, dubbed cPAL (combinatorial probe-anchor ligation) nanoballs, capillary electrophoresis, 454 pyrosequencing and mass spectrometry, often used in combination to verify variant calling. Illumina sequencers were the most commonly instruments employed in the studies whose data we used [1], [3], [4], [6], [7], [10]–[14]. The frequency of such base-call errors has been estimated at ∼0.1–0.3% before filtering, and even lower after filtering (SAMtools) [87]. Considering that sequencing errors tend to occur over long simple repeat tracts, which have low mappability, and that systematic errors at GGT were ignored (we analyzed mutations at G•C bps only), it seems unlikely that base-call errors have biased our analyses by >0.1%, an acceptable limit.

### Mappable Mutations

Approximately half of the human genome sequence comprises highly homologous repetitive DNA elements (*Alu* repeats, LINE elements etc.) and simple repeats, and an additional ∼3.6% contains Segmental Duplications, *i.e.* segments of >1 kb in length that are present at multiple loci and which share ∼90–98% sequence similarity (http://genome.ucsc.edu). Thus, because only the mappable genome may be scored for mutations, we used various methods to estimate the total number of mappable NGNN sequences to use as denominators in the *f*_i_ fractions (see below). Three methods were used for the GWS studies: 1) the entries with a mappability index of 1 (representing unique sequences) from file wgEncodeDukeMapabilityUniqueness35bp.bigWig (http://hgdownload.soe.ucsc.edu/goldenPath/hg19/database/) generated for the ENCODE project by the Duke University Institute for Genome Sciences and Policy (IGSP) and at the European Bioinformatics Institute (EBI), which we refer to as Duke35; 2) we selected sequences from the mappability file wgEncodeCrgMapabilityAlign50mer.bw ((http://hgdownload.soe.ucsc.edu/goldenPath/hg19/database/) [89] (Donohue et al., unpublished data), referred to as CRG50; and 3) we retrieved all NGNN sequences in the GRCh37/hg19 release of the human genome assembly (chromFa.tar.gz file at http://hgdownload.soe.ucsc.edu/goldenPath/hg19/bigZips/) (T_hg19).

For the EWS studies, the SureSelect Human All Exon Kit (http://www.genomics.agilent.com) was the most common platform reported [2], [4], [7], [13]. A custom RefSeq CCDS PCR primer library was used to generate the Glioblastoma dataset [5] and a set of 1,507 genes (oncogenes, tumor suppressors, “druggable targets”) were targeted in the Mixed cancer dataset [15]. Hence, three methods were used to estimate the number of mappable NGNN sequences in the EWS studies: i.e. the NGNN counts from 1) the file S0293689_Covered.bed (http://www.Agilent.com), listing the coordinates of exons targeted by the SureSelect Human All Exon Kit (AgilentV2); 2) the RefSeq exons sequences of CGR50 (CRG50_exon); and 3) the total RefSeq exons from file seq_gene.md at ftp://ftp.ncbi.nih.gov/genomes/H_sapiens/mapview/seq_gene.md.gz (*T_*exons).

### Definitions

We defined *f_i_* = *m_i_*/*t_i_*, where *m_i_* was the number of mutations at a specific NGNN•NNCN sequence (henceforth designated as NGNN) and *t_i_* was the total number of that sequence in one of the six “mappable” sets described above. The total number of NGNN sequences was doubled for the self-complementary AGCT, CGCG, GGCC and TGCA sequences since, like all NGCN sequences, they contain two mutations sites, one on the forward and one on the reverse strand. In relation to the counts of mutated NGNN motifs, if the .G.. occurred at the same genomic coordinate more than once within a cancer dataset, or if it was a homozygous mutation, it was considered as one count. Custom shell and FORTRAN scripts were used to obtain the total numbers of mappable NGNN and *f*_i_ fractions (see Text S1 for sample scripts). The normalized fractions of mutated DGNN sequences were defined as *F_i_* = *f_i_/∑f_i_*, thus, *∑F_i_* scaled to 1. N indicates any base (A/C/G/T); D indicates A/G/T; B indicates C/G/T. As mentioned, sequence designation implies double-stranded DNA (*i.e.* AGTC = (5′-AGTC-3′)•(5′-GACT-3′)). The average base stacking free energies <ΔG(ν)> were obtained from Friedman and Honig [51] by using the ΔG(ν) (ε_i_ = 2) values for the three base steps (DpG + GpN + NpN)/3. The free energy of base stacking ΔG(ν) is an estimate of the absolute contribution of base stacking to nucleic acid stability in the absence of hydrogen bonding interactions, and contains a contribution from nonpolar plus electrostatic forces, as assessed from a theoretical approach using the Amber force field and a continuum solvation model of water. The largest contribution to ΔG(ν) was found to arise from nonpolar [51], as opposed to electrostatic, interactions. Nonpolar interactions were contributed for the most part by enhancement in the Lennard-Jones component as a result of close packing, and to a smaller extent from hydrophobic interactions. Thus, the ΔG(ν) values follow the same trend as the nonpolar contributions to free energies of base stacking ΔG_np_(ν), *i.e*. purine-purine >> purine-pyrimidine > pyrimidine-purine > pyrimidine-pyrimidine, in qualitative agreement with experimental determinations [51]. The relative enrichment *E* of sequence *i*, *E_i_*, was defined as the ratio *D_i_*/*T_i_*, where *D_i_* = *d_i_*/∑*d_i_*, *d_i_* being the number of times sequence *i* was mutated at least twice at the same hg19 coordinate and *T_i_ = t_i_/∑t_i_*, *t_i_* being the total number of occurrences of sequence *i* exome-wide (T_exons). Finally, *S* = *s_n_*/∑*s_n_* and *s_n_* = *t_n_*/*c_n_*, *t_n_* being the number of times the combined *(top)* sequences were recurrently mutated and *c_n_* being the total number of NS substitutions in a particular type of cancer.

### Molecular Modeling

Three-dimensional structures of the 12 possible double-stranded DGN trinucleotides were constructed using w3DNA [90]. Hydrogen atoms, atomic charges and four neutralizing Na^+^ counterions were assigned to each sequence according to the *amber99* force field [91], using UCSF CHIMERA [92]. Na^+^ counterions were positioned next to the four DNA backbone phosphates. Each trinucleotide was energy minimized *in vacuo* using the 10,000 steps steepest descent algorithm and the *amber99* force field in GROMACS 4.5.1 [93]. Ten and 14 Å cutoffs were used for Coulomb and van der Waals interactions, respectively.

### Vertical Ionization Potentials (VIPs)

VIPs were computed using Kohn-Sham density functional theory (DFT) [94] employing the Minnesota M06-2× functional [95], [96] with all-electron 6–31G(d) basis sets [97], [98], as implemented in the GAMESS electronic structure package [99], and including backbone phosphate groups and sodium counter ions in addition to the DGN double-stranded bases. The M06-2× functional was used since this method provides accurate descriptions of hydrogen bonding and stacking interactions between base-pairs. We reasoned that the DGN set would provide the same type of information as the computationally more demanding NDGN set. Molecular orbitals were depicted using the MacMolPlt graphics program [100].

### Pathway Analysis

For individual patient samples, mutations were collated and sorted into lists of genes carrying mutations using customized R scripts (http://www.r-project.org/). The gene lists for each sample were entered into our pattern extraction pipeline analysis (PPEP) [101], as implemented in the WPS package [102], to obtain the ListHit of genes (number of genes from each list that are annotated to each pathway) for each of the BioCarta pathways. For each tumor type, each pathway was ranked on the basis of how frequently it was “hit” by individual patient samples and the ranking scores were obtained as the percentages of patient samples that had at least one hit in the corresponding pathway, using customized R scripts. The tumor type ranking scores for each pathway were combined and used to rank the pathways for all tumor types. The highest ranked pathways represent the most “popularly” hit pathways amongst all types of tumors. For each highly ranked pathway, the genes carrying the mutations were retrieved from each patient sample, ranked and displayed as gene-level heatmaps. For the pathway analysis of recurrent NS substitutions, the relevant genes for each tumor type were collated into lists and subjected to PPEP analysis, as described above.

### Hierarchical Clustering

Agglomerative hierarchical clustering dendrograms [103] were built using either the regression coefficients, *r*, between the fractions of mutated DGN sequences, *f*(DGN), and the VIP values, or the absolute orthogonal distances (Manhattan distances) between each *f*(NGNN) data point for all datasets. All-to-all comparisons were performed, allowing the relative estimation of all components of the systems, including the reference VIP branch.

## Supporting Information

Figure S1 Individual samples from the same cancer dataset and cancer types share mutational patterns. *Panel A*, analysis of variance for the normalized fractions of CGNN *vs*. DGNN and DGRN *vs*. DGYN for the 14 Melanoma_ews samples computed from the AgilentV2 mappability counts; *full circles*, Holm-Sidak test on the difference of means (normality test by Shapiro-Wilk, P = 0.657); *triangles*, Kruskal-Wallis one-way ANOVA on ranks (H = 18.66). *Panel B*, hierarchical clustering of the 20 largest datasets (Table 1) plus 5 individual samples comprising the Pancreatic_ca dataset, computed from the sum of the absolute difference (Manhattan distance) in the scaled frequencies of mutated NGN trinucleotides obtained by summing the 4^th^ positions of the NGNN frequencies. Mutated frequencies for the NGNN sequences were according to Duke35 (GWS) and AgilentV2 (EWS) counts.
(TIF)

Figure S2 Plots of the correlation between f(DGN) and VIPs. *x*-axis, normalized mutations fractions; *y*-axis, VIP values; *Panel A*, *f*_i_ for Melanoma_ews computed using the AgilentV2 mappability counts; *Panels B* and *C*, *f*_i_ for the Melanoma_gws and Liver_riken, respectively, computed using the Duke35 mappability counts; *Panel D*, averaged *f*_i_ values for 18 cancer datasets (Table S6). See Legend to Fig. 1 for details.
(TIF)

Figure S3 Genes with NS substitutions and mutation spectrum. *Panel A*. Plot of the number of NS substitutions per 100 amino acids (*H*) for the 29 genes in the combined cancer datasets with ≥24 NS substitutions. *Panel B*, as in Panel A, for genes with ≥4 recurrent NS substitutions; *dotted line*, median protein size in amino acid residues, as derived from http://www.genecards.org/. *Panel C*, percentage of the six possible types of SBS for the 35,480 NS substitutions in the combined cancer genomes.
(TIF)

Figure S4 Pathway-level heatmaps. *Panel A*, pathway-level heatmap for the highest-ranking (from top down) pathways (www.biocarta.com) based on the “fractional representation” of genes hit by NS substitutions across all combined individual patient samples for all tumor types. *Rows*, pathways; *columns*: tumor types; *Fractional representation*, color-gradient of the fraction of patient samples in each tumor type that have at least one gene-hit in the corresponding pathway. *Panel B*, patient sample-specific pathway-level heatmap. Gene-level heatmap displaying the individual patient samples (*rows*) carrying NS substitutions in genes belonging to a common pathway (*columns*) ranked in order of their “popularity” for the top pathways shown in Panel A. *Panel C*, Telomeres-Aging term gene-level heatmap. The detailed gene-level heatmap for the top pathway “Telomeres-Aging” shown in Panel B. *Columns*: individual patient samples arranged by tumor types; *rows*: genes hit by NS substitutions ranked in order of number of mutations.
(TIF)

Figure S5 Nucleotide conservation and variation at splice junctions. *Top panel*, fractional conservation within +/−25 nt in 295,093 unique donor and acceptor splice junctions from annotated genes in the GRCh37/hg19 human genome assembly. *Middle panel*, number of single nucleotide variants (SNV) in the “rs” set of the 1000 Genomes Project, mapped as shown in the top panel. *Bottom panel*, number of SBSs in the TCGARN cancer dataset mapping to the splice junctions shown on the top panel.
(TIF)

Table S1 NGNN counts and relative abundance in the whole genome and the mappable genome. *Panel A*, whole genome; *Panel B*, exome; *raw NGNN counts*, number of NGNN sequences in each set; *relative NGNN percentage*, raw numbers divided by total ×100; *grouping*, relative percentages for CGNN, DGNN, NGNN containing at least 3 guanines (3G), DGRN and DGYN; *comparison between sets*, values obtained from setA/setB*100-100.
(XLSX)

Table S2 Statistical significance of NGNN representation in mappable genome and exome. Two-tailed *t*-tests for the “*comparison between sets*” data from Table S1.
(DOCX)

Table S3 List of chromosomal positions (GRCh37/hg19) affected by SBSs in the cancer datasets. Only non-redundant positions within each dataset are recorded.
(XLSX)

Table S4 f_i_ fractions and associated statistics. *Panel A*, counts of SBSs occurring at each NGNN sequence in all datasets and ratios of SBSs affecting G•C base-pairs; *Panel B*, *f*_i_ fractions for the GWS datasets according to three methods and associated statistics (two-tailed paired *t*-test); *Panel C*, *f*_i_ fractions for the EWS datasets according to three methods and associated statistics (two-tailed paired *t*-test); *Panel D*, *f*_i_ fractions for the 2 small EWS datasets (Colorectal_1 and Colorectal_2) and the 14 Melanoma_ews samples according to three methods and associated statistics (*z*-test; P(α)_0.05_ statistics); *Panel E*, chromosomal positions and counts of SBSs occurring at each NGNN sequence in the 14 patient samples from the Melanoma_ews dataset (*T series) and in the 5 patient samples from the Pancreatic_ca dataset (PCSI* series).
(XLSX)

Table S5 f_i_ fractions for NGNN sequences with P3-A or P3-B (C/G/T) in melanomas. Statistical analyses, two-tailed *t*-tests.
(DOCX)

Table S6 Normalized mutation fractions at NGRN sequences in melanoma. EWS or GWS *f*(NGRN) fractions within each dataset were normalized by dividing for *f*(CGAA), which displayed the highest value in all datasets. *f*_i_ fractions were computed using the T_hg19 and T_exons counts. *Green background*, the 8 combinations of the NGRA sequence; *tan background*, *f*(NGRB) values that were lower than the corresponding *f*(NGRA) values; *turquoise background*, *f*(NGRB) values that were higher than the corresponding *f*(NGRA) values; *Wei*, [10]; *Pleasance*, [11]; *Berger*, [17]; *Nikolaev*, [21]; *Stark*, [40]; *Krauthammer*, [34].
(DOCX)

Table S7 Correlations with free energy of base stacking and VIPs. *Panel A*, correlations of *ln*[*f*(DGNN)] values with free energies of base stacking; *Panel B*, correlations of *ln*[*f*(DGN)] values with VIPs. For the combined datasets, the fractions were normalized. *f*_i_ values were computed using the Duke35 (GWS) and AgilentV2 (EWS) mappability counts.
(DOCX)

Table S8 Fractional representation of genes with NS substitutions across cancer tissue types and their Aggregate Score. List of genes found to contain non-synonymous substitutions and their combined fractional representation, expressed as Aggregate Score, across cancer types.
(XLSX)

Table S9 Recurrent NS substitutions and driver mutations. Hg19 coordinates containing the largest number of recurrent NS substitutions for various types of motifs and their potential as driver mutations; ^1^ sequences are reported with the mutated base (A or G) at P2; ^2^ support from PubMed for a driver mutation following searches for “gene_name & mutated_codon” or “gene_name & cancer” and a manual review of the articles found: *** mutated codon that has been reported as a driver mutation; ** (!) mutated codon in p53 that has not been reported to harbor a driver mutation; ** strong support for gene mutation or change in gene expression being involved in tumorigenesis; * weak support for gene mutation or change in gene expression being involved in tumorigenesis; *unk*, insufficient information to assess whether a gene mutation or change in gene expression was involved in tumorigenesis.
(DOCX)

Table S10 Individual patient samples harboring genes with ≥4 NS substitutions. Sequence context-dependency of patient samples which experienced ≥4 NS substitutions in the same gene. *White background*, SBSs at DGNN sequences; *white on black background*, SBSs at CGNN sequences; *gray background*, SBSs at NANN sequences; *bold*, SBSs at DGRN sequences. Fraction of NGNN sequences: 30/35 = 86%).
(DOCX)

Text S1 Supporting information. The text contains information on the distribution of NGNN sequences in the mappability files and in Segmental Duplications, a description of mutational mechanisms shared by similar cancer types and individual samples, the analysis of recurrent non-synonymous substitutions, and exemplary scripts for obtaining the fractions *f*_i_ of mutated NGNN sequences.
(DOCX)
