# Supplementary figures and images for: Guanine Holes Are Prominent Targets for Mutation in Cancer and Inherited Disease
Source: PLoS Genet. 2013 Sep 26;9(9):e1003816. doi: 10.1371/journal.pgen.1003816 (PMC3784513; doi:10.1371/journal.pgen.1003816)

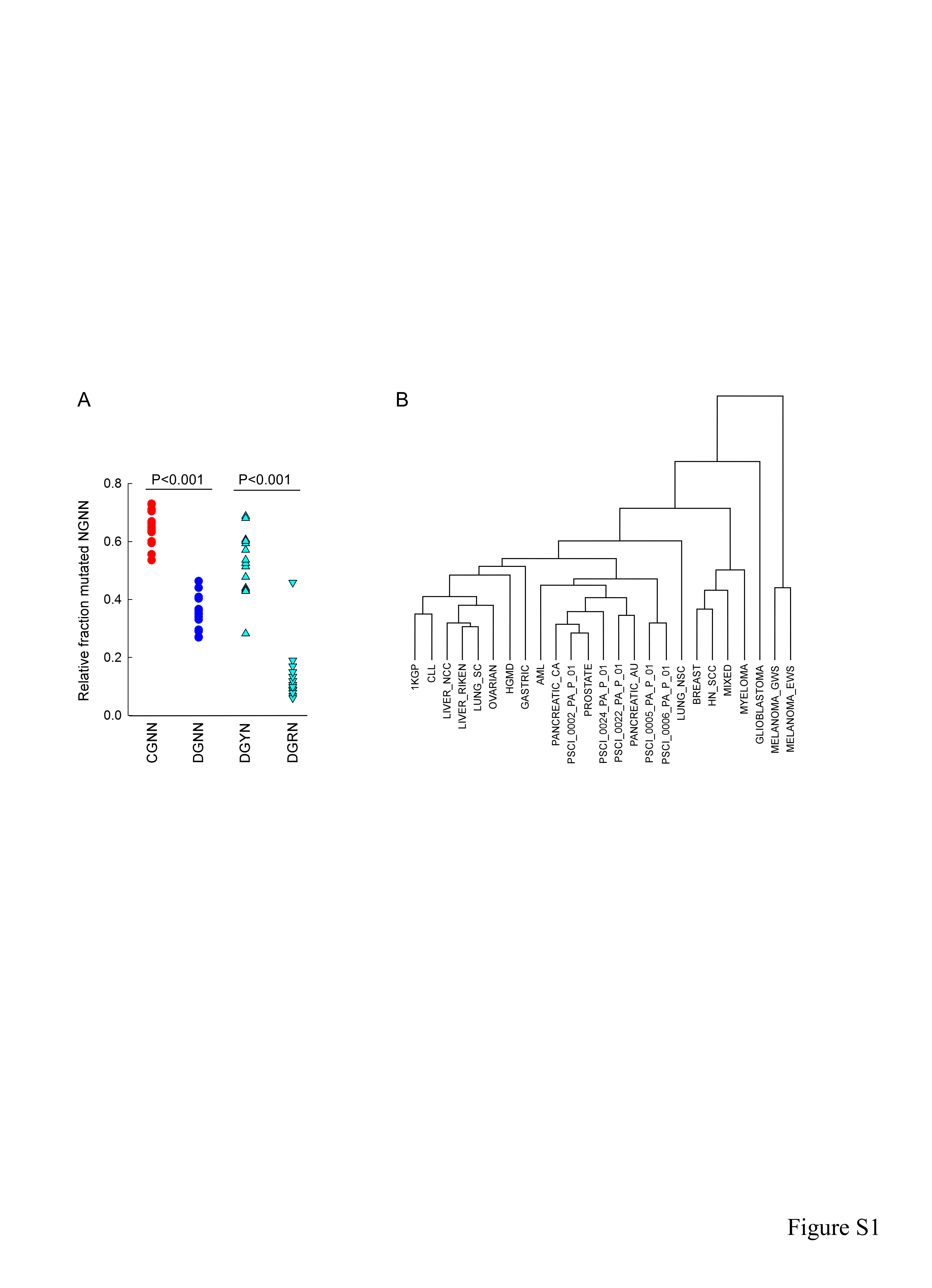

Supplement: Figure S1 — Individual samples from the same cancer dataset and cancer types share mutational patterns. Panel A, analysis of variance for the normalized fractions of CGNN vs. DGNN and DGRN vs. DGYN for the 14 Melanoma_ews samples computed from the AgilentV2 mappability counts; full circles, Holm-Sidak test on the difference of means (normality test by Shapiro-Wilk, P = 0.657); triangles, Kruskal-Wallis one-way ANOVA on ranks (H = 18.66). Panel B, hierarchical clustering of the 20 largest datasets (Table 1) plus 5 individual samples comprising the Pancreatic_ca dataset, computed from the sum of the absolute difference (Manhattan distance) in the scaled frequencies of mutated NGN trinucleotides obtained by summing the 4th positions of the NGNN frequencies. Mutated frequencies for the NGNN sequences were according to Duke35 (GWS) and AgilentV2 (EWS) counts. (TIF) [file pgen.1003816.s001.tif]

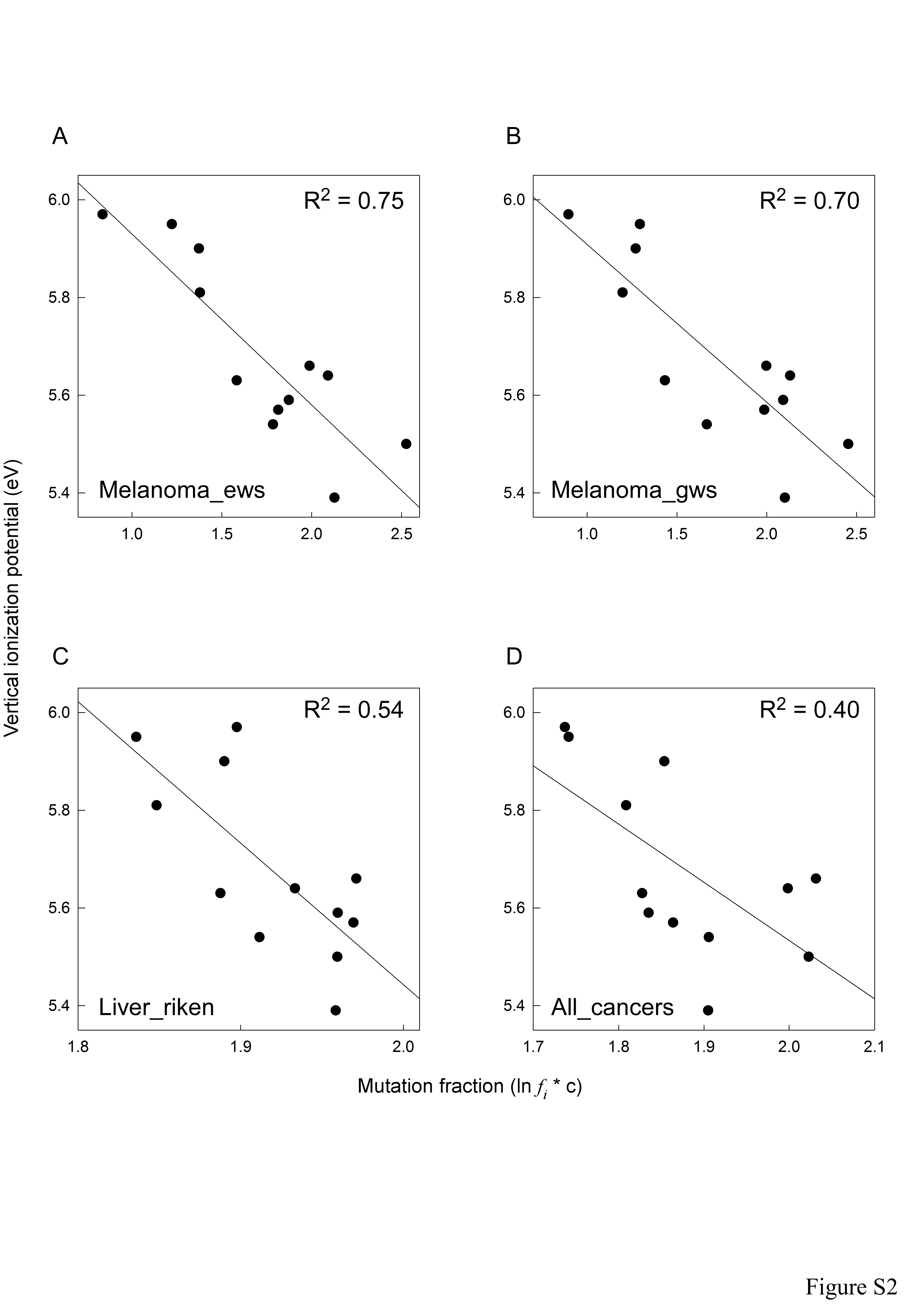

Supplement: Figure S2 — Plots of the correlation between f(DGN) and VIPs. x-axis, normalized mutations fractions; y-axis, VIP values; Panel A, f i for Melanoma_ews computed using the AgilentV2 mappability counts; Panels B and C, f i for the Melanoma_gws and Liver_riken, respectively, computed using the Duke35 mappability counts; Panel D, averaged f i values for 18 cancer datasets (Table S6). See Legend to Fig. 1 for details. (TIF) [file pgen.1003816.s002.tif]

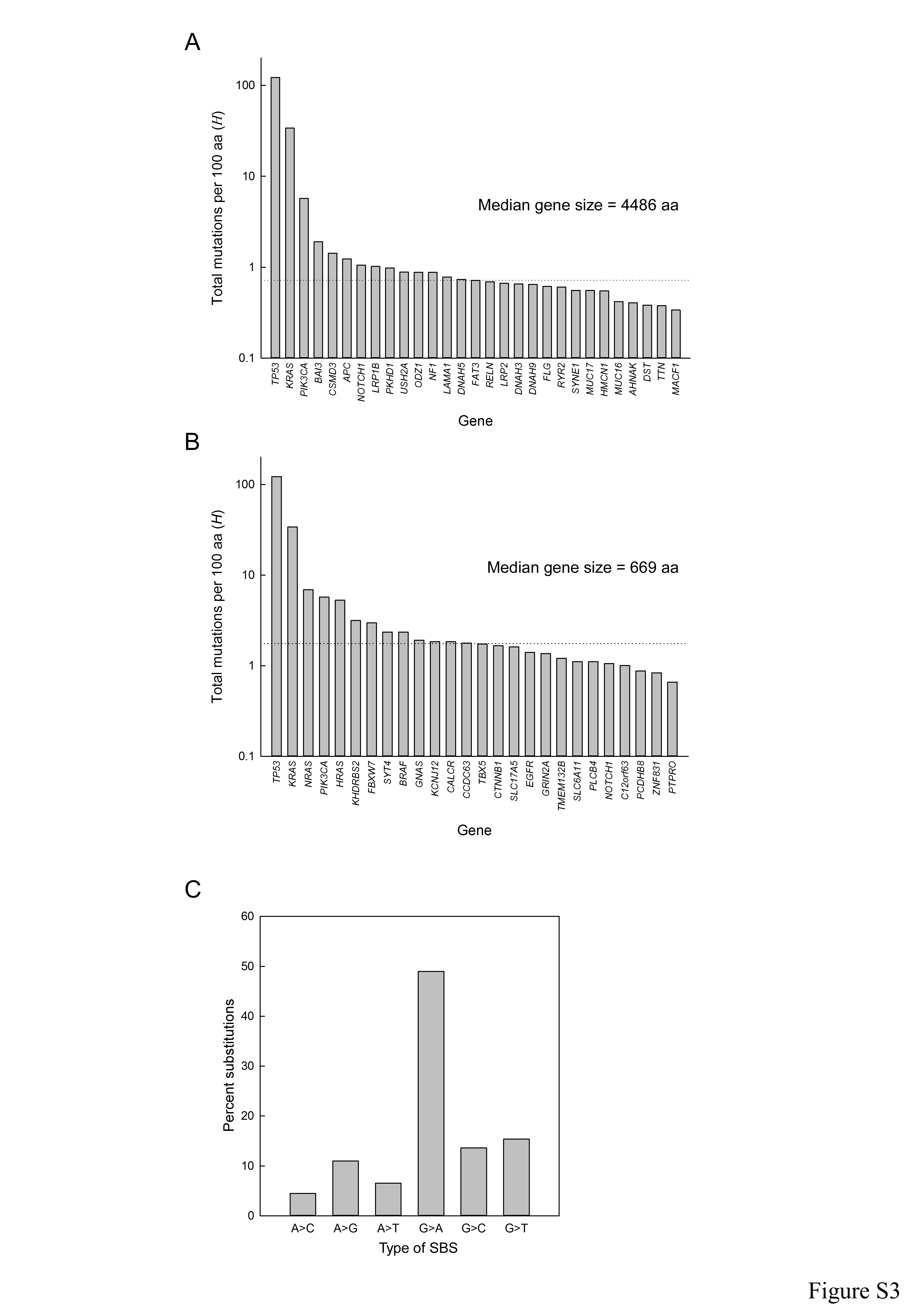

Supplement: Figure S3 — Genes with NS substitutions and mutation spectrum. Panel A. Plot of the number of NS substitutions per 100 amino acids (H) for the 29 genes in the combined cancer datasets with ≥24 NS substitutions. Panel B, as in Panel A, for genes with ≥4 recurrent NS substitutions; dotted line, median protein size in amino acid residues, as derived from http://www.genecards.org/. Panel C, percentage of the six possible types of SBS for the 35,480 NS substitutions in the combined cancer genomes. (TIF) [file pgen.1003816.s003.tif]

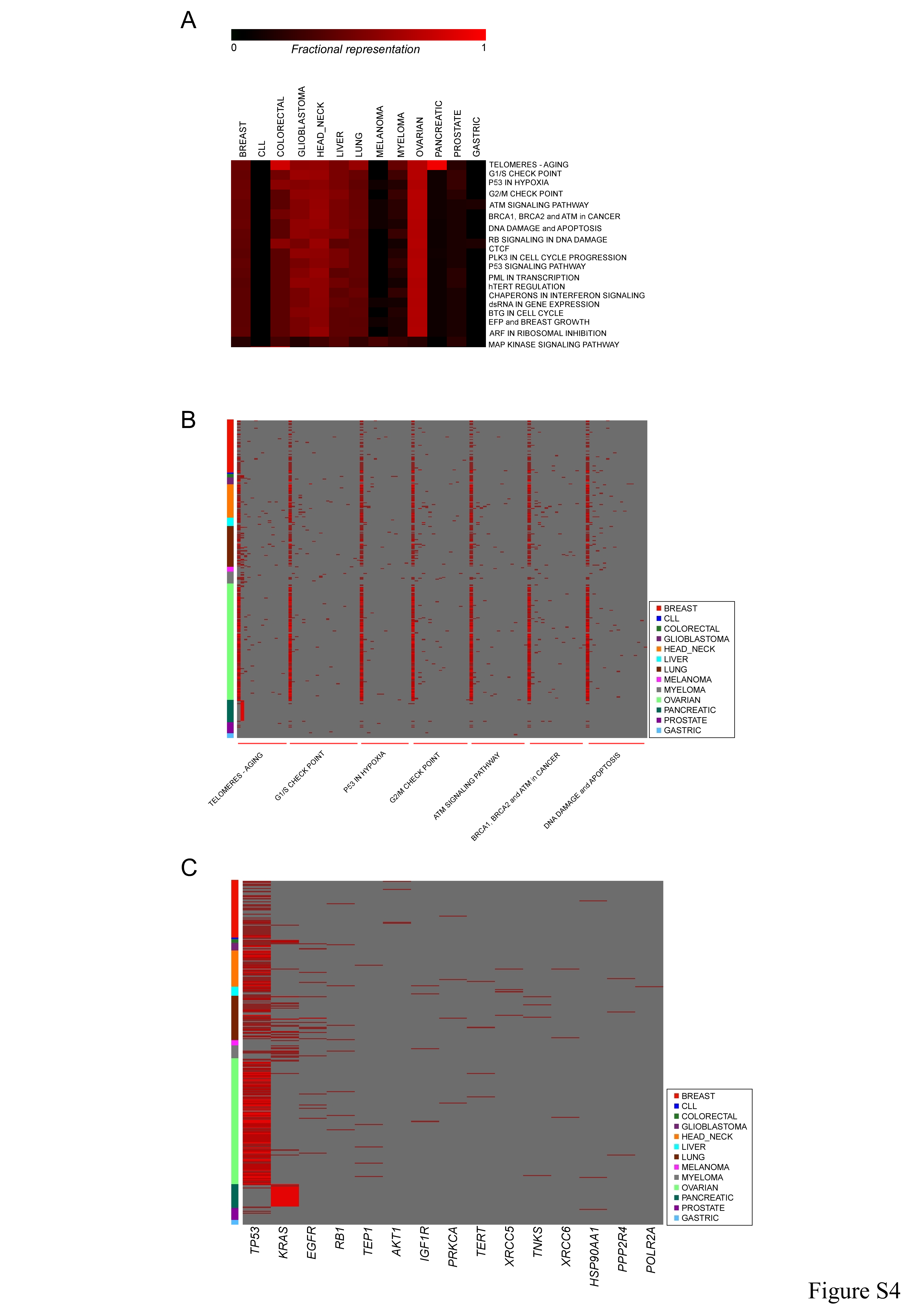

Supplement: Figure S4 — Pathway-level heatmaps. Panel A, pathway-level heatmap for the highest-ranking (from top down) pathways (www.biocarta.com) based on the “fractional representation” of genes hit by NS substitutions across all combined individual patient samples for all tumor types. Rows, pathways; columns: tumor types; Fractional representation, color-gradient of the fraction of patient samples in each tumor type that have at least one gene-hit in the corresponding pathway. Panel B, patient sample-specific pathway-level heatmap. Gene-level heatmap displaying the individual patient samples (rows) carrying NS substitutions in genes belonging to a common pathway (columns) ranked in order of their “popularity” for the top pathways shown in Panel A. Panel C, Telomeres-Aging term gene-level heatmap. The detailed gene-level heatmap for the top pathway “Telomeres-Aging” shown in Panel B. Columns: individual patient samples arranged by tumor types; rows: genes hit by NS substitutions ranked in order of number of mutations. (TIF) [file pgen.1003816.s004.tif]

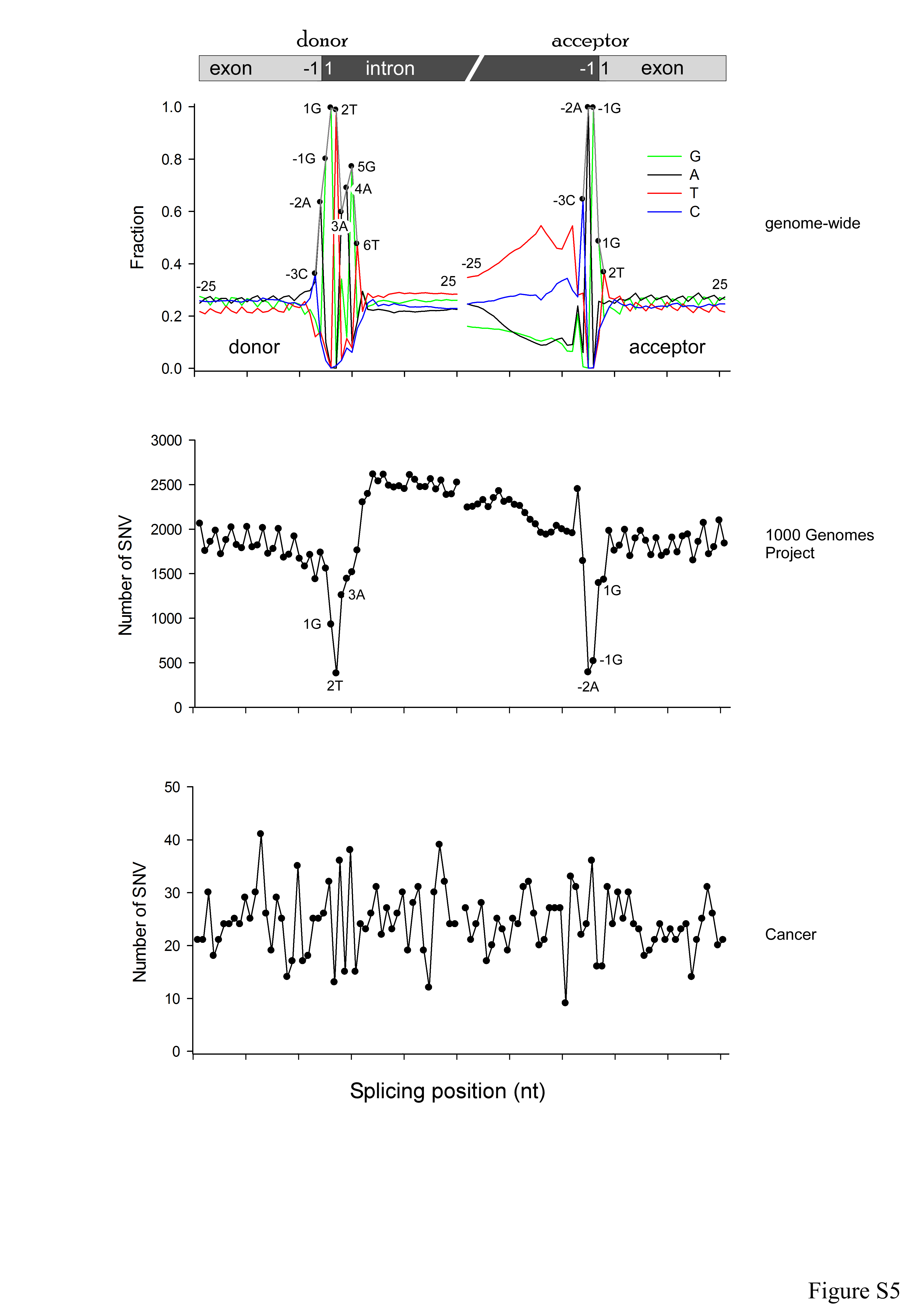

Supplement: Figure S5 — Nucleotide conservation and variation at splice junctions. Top panel, fractional conservation within +/−25 nt in 295,093 unique donor and acceptor splice junctions from annotated genes in the GRCh37/hg19 human genome assembly. Middle panel, number of single nucleotide variants (SNV) in the “rs” set of the 1000 Genomes Project, mapped as shown in the top panel. Bottom panel, number of SBSs in the TCGARN cancer dataset mapping to the splice junctions shown on the top panel. (TIF) [file pgen.1003816.s005.tif]
