# Supplementary material for: Guanine Holes Are Prominent Targets for Mutation in Cancer and Inherited Disease
Source: PLoS Genet. 2013 Sep 26;9(9):e1003816. doi: 10.1371/journal.pgen.1003816 (PMC3784513; doi:10.1371/journal.pgen.1003816)
Supplement: Table S2 — Statistical significance of NGNN representation in mappable genome and exome. Two-tailed t-tests for the “comparison between sets” data from Table S1. (DOCX) [file pgen.1003816.s007.docx]

**Table S2.** *Statistical significance of* *NGNN representation in mappable genome and exome*

| Comparison | Tetra set | Mean | S.D. | Paired t test | Pair set |
| --- | --- | --- | --- | --- | --- |
| Duke35 *vs.* T_hg19 | CGNN | -6.19 | 7.60 | 7.20e-05 | (DGNN) |
|  | DGNN | 0.18 | 3.73 |  |  |
|  | 3G | -2.57 | 3.90 | 0.019 | (DGNN) |
|  | DGRN | 0.07 | 3.84 |  |  |
|  | DGYN | 0.33 | 3.68 | 0.826 | (DGRN) |
| CRG50 *vs.* T_hg19 | CGNN | -9.79 | 9.32 | 1.36e-06 | (DGNN) |
|  | DGNN | 0.26 | 4.79 |  |  |
|  | 3G | -3.32 | 4.81 | 0.016 | (DGNN) |
|  | DGRN | 0.18 | 4.86 |  |  |
|  | DGYN | 0.38 | 4.82 | 0.895 | (DGRN) |
| SD *vs*. Hg19noSD | CGNN | 19.69 | 8.45 | 1.03e-14 | (DGNN) |
|  | DGNN | -0.24 | 5.64 |  |  |
|  | 3G | 5.90 | 3.47 | 2.34e-04 | (DGNN) |
|  | DGRN | 0.52 | 5.23 |  |  |
|  | DGYN | -1.24 | 6.16 | 0.325 | (DGRN) |
| RM *vs*. Hg19noRM | CGNN | 2.66 | 24.76 | 0.711 | (DGNN) |
|  | DGNN | 0.73 | 14.15 |  |  |
|  | 3G | 4.35 | 18.15 | 0.433 | (DGNN) |
|  | DGRN | -0.22 | 16.21 |  |  |
|  | DGYN | 1.99 | 11.17 | 0.623 | (DGRN) |
| AgilentV2 *vs*. | CGNN | 1.39 | 7.91 | 0.645 | (DGNN) |
| T_exons | DGNN | 0.27 | 8.35 |  |  |
|  | 3G | 2.03 | 3.76 | 0.435 | (DGNN) |
|  | DGRN | 1.15 | 6.23 |  |  |
|  | DGYN | -0.91 | 10.62 | 0.436 | (DGRN) |
| CGR50_exons *vs.* | CGNN | 0.82 | 1.08 | 1.77e-03 | (DGNN) |
| T_exons | DGNN | -0.12 | 0.94 |  |  |
|  | 3G | -0.16 | 1.21 | 0.915 | (DGNN) |
|  | DGRN | -0.12 | 1.08 |  |  |
|  | DGYN | -0.13 | 0.73 | 0.962 | (DGRN) |
