# Supplementary material for: Guanine Holes Are Prominent Targets for Mutation in Cancer and Inherited Disease
Source: PLoS Genet. 2013 Sep 26;9(9):e1003816. doi: 10.1371/journal.pgen.1003816 (PMC3784513; doi:10.1371/journal.pgen.1003816)
Supplement: Table S5 — fi fractions for NGNN sequences with P3-A or P3-B (C/G/T) in melanomas. Statistical analyses, two-tailed t-tests. (DOCX) [file pgen.1003816.s010.docx]

**Table S5.** *f*_i_ *fractions for NGNN sequences with P3-A or P3-B (C/G/T) in melanomas*

| Sequence | Melanoma_gws | | |  | Melanoma_ews | | |
| --- | --- | --- | --- | --- | --- | --- | --- |
|  | Mean (SD)  (x 10^-5^) | | P-value  t-test |  | Mean (SD)  (x 10^-5^) | | P-value  t-test |
|  |  |  |  |  |  |  |  |
| CGAN | 14.28 (10.67) | |  |  | 68.38 (35.82) | |  |
| CGBN | 1.74 (1.79) | | 9.115 x 10^-4^ |  | 6.39 (5.81) | | 2.375 x 10^-5^ |
|  |  |  |  |  |  |  |  |
| DGAN | 4.94 (4.02) | |  |  | 20.50 (17.51) | |  |
| DGBN | 1.51 (1.65) | | 1.123 x 10^-4^ |  | 5.45 (5.26) | | 2.838 x 10^-5^ |
|  |  |  |  |  |  |  |  |
| CGAN *vs*. DGAN |  |  | 0.019 |  |  |  | 2.621 x 10^-3^ |
| CGBN *vs*. DGBN |  |  | 0.697 |  |  |  | 0.604 |
|  |  |  |  |  |  |  |  |
