# Supplementary material for: Guanine Holes Are Prominent Targets for Mutation in Cancer and Inherited Disease
Source: PLoS Genet. 2013 Sep 26;9(9):e1003816. doi: 10.1371/journal.pgen.1003816 (PMC3784513; doi:10.1371/journal.pgen.1003816)
Supplement: Table S6 — Normalized mutation fractions at NGRN sequences in melanoma. EWS or GWS f(NGRN) fractions within each dataset were normalized by dividing for f(CGAA), which displayed the highest value in all datasets. f i fractions were computed using the T_hg19 and T_exons counts. Green background, the 8 combinations of the NGRA sequence; tan background, f(NGRB) values that were lower than the corresponding f(NGRA) values; turquoise background, f(NGRB) values that were higher than the corresponding f(NGRA) values; Wei, [10]; Pleasance, [11]; Berger, [17]; Nikolaev, [21]; Stark, [40]; Krauthammer, [34]. (DOCX) [file pgen.1003816.s011.docx]

**Table S6.** *Normalized mutation fractions at NGRN sequences in melanoma*

| Sequence | Wei | Pleasance | Berger | Nikolaev | Stark | Krauthammer |
| --- | --- | --- | --- | --- | --- | --- |
| AGAA | 0.1691 | 0.1884 | 0.1324 | 0.1410 | 0.1066 | 0.1034 |
| AGAC | 0.0528 | 0.0557 | 0.0836 | 0.0766 | 0.0391 | 0.0615 |
| AGAG | 0.1139 | 0.1040 | 0.0990 | 0.1063 | 0.1204 | 0.0929 |
| AGAT | 0.0686 | 0.0977 | 0.1195 | 0.0916 | 0.0765 | 0.0764 |
|  |  |  |  |  |  |  |
| AGGA | 0.1141 | 0.1664 | 0.1160 | 0.1943 | 0.1287 | 0.1073 |
| AGGC | 0.0444 | 0.0436 | 0.0445 | 0.0792 | 0.0542 | 0.0372 |
| AGGG | 0.0754 | 0.1376 | 0.0770 | 0.0998 | 0.0775 | 0.0552 |
| AGGT | 0.0517 | 0.0625 | 0.1002 | 0.0839 | 0.0928 | 0.0679 |
|  |  |  |  |  |  |  |
| CGAA | 1.0000 | 1.0000 | 1.0000 | 1.0000 | 1.0000 | 1.0000 |
| CGAC | 0.3465 | 0.2020 | 0.2983 | 0.2018 | 0.2706 | 0.2462 |
| CGAG | 0.3515 | 0.1694 | 0.3327 | 0.2276 | 0.3634 | 0.3167 |
| CGAT | 0.9242 | 0.6247 | 0.6846 | 0.9076 | 0.8856 | 0.6796 |
|  |  |  |  |  |  |  |
| CGGA | 0.1952 | 0.1875 | 0.1795 | 0.2227 | 0.3557 | 0.2170 |
| CGGC | 0.0641 | 0.0361 | 0.0803 | 0.0412 | 0.0550 | 0.0481 |
| CGGG | 0.1008 | 0.1699 | 0.0977 | 0.1194 | 0.0735 | 0.0704 |
| CGGT | 0.0888 | 0.0742 | 0.0891 | 0.1089 | 0.1272 | 0.0733 |
|  |  |  |  |  |  |  |
| GGAA | 0.6034 | 0.5982 | 0.5188 | 0.6288 | 0.6282 | 0.5647 |
| GGAC | 0.1706 | 0.1396 | 0.1983 | 0.2175 | 0.2665 | 0.1900 |
| GGAG | 0.2365 | 0.1758 | 0.2652 | 0.2899 | 0.2776 | 0.2699 |
| GGAT | 0.3787 | 0.2964 | 0.3580 | 0.3898 | 0.3557 | 0.3593 |
|  |  |  |  |  |  |  |
| GGGA | 0.2373 | 0.2304 | 0.2149 | 0.2655 | 0.2471 | 0.2204 |
| GGGC | 0.0665 | 0.0651 | 0.0878 | 0.1047 | 0.1151 | 0.0653 |
| GGGG | 0.1266 | 0.1848 | 0.1643 | 0.1686 | 0.1490 | 0.1207 |
| GGGT | 0.1234 | 0.0789 | 0.1110 | 0.1152 | 0.1422 | 0.0885 |
|  |  |  |  |  |  |  |
| TGAA | 0.1745 | 0.2629 | 0.1798 | 0.2499 | 0.2447 | 0.2149 |
| TGAC | 0.0404 | 0.0413 | 0.0520 | 0.0424 | 0.0315 | 0.0356 |
| TGAG | 0.0536 | 0.0575 | 0.0603 | 0.0644 | 0.1084 | 0.0538 |
| TGAT | 0.2325 | 0.2229 | 0.1757 | 0.1843 | 0.1697 | 0.1719 |
|  |  |  |  |  |  |  |
| TGGA | 0.1485 | 0.1918 | 0.1170 | 0.1629 | 0.1830 | 0.1760 |
| TGGC | 0.0307 | 0.0389 | 0.0314 | 0.0564 | 0.0282 | 0.0315 |
| TGGG | 0.0582 | 0.1914 | 0.0767 | 0.0826 | 0.0779 | 0.0669 |
| TGGT | 0.0740 | 0.0819 | 0.0547 | 0.0605 | 0.0736 | 0.0568 |
