# Supplementary material for: Guanine Holes Are Prominent Targets for Mutation in Cancer and Inherited Disease
Source: PLoS Genet. 2013 Sep 26;9(9):e1003816. doi: 10.1371/journal.pgen.1003816 (PMC3784513; doi:10.1371/journal.pgen.1003816)
Supplement: Table S7 — Correlations with free energy of base stacking and VIPs. Panel A, correlations of ln[f(DGNN)] values with free energies of base stacking; Panel B, correlations of ln[f(DGN)] values with VIPs. For the combined datasets, the fractions were normalized. f i values were computed using the Duke35 (GWS) and AgilentV2 (EWS) mappability counts. (DOCX) [file pgen.1003816.s012.docx]

**Table S7.** *Correlations with free energy of base stacking and VIPs*

A – Correlations of *ln*[*f*(DGNN)] values with base stacking energies

| Dataset | R^2^ | P-value | P(alpha) |
| --- | --- | --- | --- |
| Melanoma_gws | 0.709 | <0.001 | 1.000 |
| Melanoma_ews | 0.644 | <0.001 | 1.000 |
| Lung_nsc | 0.382 | <0.001 | 0.998 |
| Liver_riken | 0.217 | <0.001 | 0.923 |
| Mixed | 0.097 | 0.031 | 0.579 |
| HN_SCC | 0.068 | 0.074 | 0.433 |
| Breast | 0.040 | 0.173 | 0.274 |
| *All 7 datasets* | *0.472* | *<0.001* | *1.000* |

B – Correlations of *ln*[*f*(DGN)] values with VIPs

| Dataset | R^2^ | P-value | P(alpha) |
| --- | --- | --- | --- |
| Melanoma_ews | 0.750 | <0.001 | 0.977 |
| Melanoma_gws | 0.697 | <0.001 | 0.951 |
| Lung_nsc | 0.678 | <0.001 | 0.939 |
| Liver_riken | 0.538 | 0.007 | 0.802 |
| GBM | 0.287 | 0.073 | 0.434 |
| Mixed | 0.160 | 0.197 | 0.246 |
| CLL | 0.113 | 0.285 | 0.181 |
| HGMD | 0.076 | 0.385 | 0.134 |
| Pancreatic_au | 0.053 | 0.473 | 0.104 |
| Liver_ncc | 0.051 | 0.481 | 0.102 |
| AML | 0.045 | 0.506 | 0.095 |
| HN_SCC | 0.045 | 0.507 | 0.095 |
| Breast | 0.031 | 0.587 | 0.076 |
| Prostate | 0.026 | 0.618 | 0.070 |
| Gastric | 0.024 | 0.634 | 0.067 |
| Ovarian | 0.013 | 0.723 | 0.053 |
| Pancreatic_ca | 0.006 | 0.812 | 0.042 |
| Lung_sc | 0.004 | 0.841 | 0.039 |
| 1KGP | 0.002 | 0.898 | 0.033 |
| Myeloma | 0.001 | 0.914 | 0.032 |
| *All 18 cancer datasets* | *0.404* | *0.026* | *0.615* |
