# Supplementary material for: Guanine Holes Are Prominent Targets for Mutation in Cancer and Inherited Disease
Source: PLoS Genet. 2013 Sep 26;9(9):e1003816. doi: 10.1371/journal.pgen.1003816 (PMC3784513; doi:10.1371/journal.pgen.1003816)
Supplement: Table S9 — Recurrent NS substitutions and driver mutations. Hg19 coordinates containing the largest number of recurrent NS substitutions for various types of motifs and their potential as driver mutations; 1 sequences are reported with the mutated base (A or G) at P2; 2 support from PubMed for a driver mutation following searches for “gene_name & mutated_codon” or “gene_name & cancer” and a manual review of the articles found: *** mutated codon that has been reported as a driver mutation; ** (!) mutated codon in p53 that has not been reported to harbor a driver mutation; ** strong support for gene mutation or change in gene expression being involved in tumorigenesis; * weak support for gene mutation or change in gene expression being involved in tumorigenesis; unk, insufficient information to assess whether a gene mutation or change in gene expression was involved in tumorigenesis. (DOCX) [file pgen.1003816.s014.docx]

**Table S9.** *Recurrent NS substitutions and driver mutations*

|  |  | |  |  | |  |
| --- | --- | --- | --- | --- | --- | --- |
| hg19 coordinate | # cases | Sequence  (# cases) ^1^ | Gene | Codon | Driver potential ^2^ | Cancer type  (# cases) |
|  |  |  |  |  |  |  |
| ***A. hg19 coordinates with >12 rNS substitutions*** | | | | | | |
|  | | | | | | |
| chr3:178952085 | 41 | C**A**TC | *PIK3CA* | p.H1047R | *** | Breast (20) |
|  |  |  |  |  |  | Head_Neck (3) |
|  |  |  |  |  |  | Colorectal (2) |
|  |  |  |  |  |  | Ovarian (2) |
|  |  |  |  |  |  | Gastric (1) |
|  |  |  |  |  |  | Liver (1) |
|  |  |  |  |  |  | Prostate (1) |
|  |  |  |  | p.H1047L | *** | Breast (6) |
|  |  |  |  |  |  | Head_Neck (5) |
| chr12:25398284/5 | 41 | G**G**TG (27) | *KRAS* | p.G12D | *** | Pancreatic (4) |
|  |  |  |  |  |  | Colorectal (4) |
|  |  |  |  |  |  | Lung (3) |
|  |  |  |  |  |  | Breast (1) |
|  |  |  |  |  |  | Gastric (1) |
|  |  |  |  | p.G12V | *** | Ovary (4) |
|  |  |  |  |  |  | Colorectal (3) |
|  |  |  |  |  |  | Lung (3) |
|  |  |  |  |  |  | Pancreatic (3) |
|  |  |  |  | p.G12A | *** | Colorectal (1) |
|  |  | T**G**GT (14) |  | p.G12C | *** | Lung (11) |
|  |  |  |  | p.G12R | *** | Ovarian (1) |
|  |  |  |  |  |  | Pancreatic (1) |
|  |  |  |  | p.G12S | *** | Colorectal (1) |
| chr17:7577120/1 | 34 | C**G**TG (20) | *TP53* | p.R273H | *** | Ovary (12) |
|  |  |  |  |  |  | Head_Neck (2) |
|  |  |  |  |  |  | Colorectal (1) |
|  |  |  |  |  |  | Glioblastoma (1) |
|  |  |  |  | p.R273L | *** | Ovary (2) |
|  |  |  |  |  |  | Breast (1) |
|  |  |  |  | p.R273P | *** | Ovary (1) |
|  |  | C**G**CA (14) |  | p.R273C | *** | Ovary (8) |
|  |  |  |  |  |  | Head_Neck (4) |
|  |  |  |  |  |  | Colorectal (1) |
|  |  |  |  |  |  | Glioblastoma (1) |
| chr17:7577538/9 | 28 | C**G**GA (15) | *TP53* | p.R248Q | *** | Ovary (9) |
|  |  |  |  |  |  | Head_Neck (4) |
|  |  |  |  |  |  | Breast (2) |
|  |  | C**G**GT (13) |  | p.R248W | *** | Ovary (9) |
|  |  |  |  |  |  | Breast (1) |
|  |  |  |  |  |  | Gastric (1) |
|  |  |  |  |  |  | Glioblastoma (1) |
|  |  |  |  | p.R248G | ** (!) | Ovary (1) |
| chr17:7578190 | 19 | T**A**TG | *TP53* | p.Y220C | *** | Ovary (16) |
|  |  |  |  |  |  | Head_Neck (3) |
| chr17:7578406 | 18 | C**G**CT | *TP53* | p.R175H | *** | Ovary (8) |
|  |  |  |  |  |  | Head_Neck (7) |
|  |  |  |  |  |  | Breast (1) |
|  |  |  |  |  |  | Gastric (1) |
|  |  |  |  |  |  | Pancreatic (1) |
| chr7:140453136 | 14 | C**A**CT | *BRAF* | p.V600E | *** | Melanoma (8) |
|  |  |  |  |  |  | Ovary (4) |
|  |  |  |  |  |  | Breast (1) |
|  |  |  |  |  |  | Colorectal (1) |
|  |  |  |  |  |  |  |
| ***B. CGNN sequences most often mutated in rNS substitutions*** | | | | | | |
|  | | | | | | |
| chr1:117122285 | 35 | C**G**TC | *IGSF3* | p.D1021E | * | Myeloma (2) |
| chr1:24664220 |  |  | *GRHL3* | p.V266I | ** | Ovary (1) |
|  |  |  |  |  |  | Liver (1) |
| chr11:88337977 |  |  | *GRM5* | p.R435W | ** | Head_Neck (1) |
|  |  |  |  |  |  | Prostate (1) |
| chr5:7802364 |  |  | *ADCY2* | p.V888I | ** | Ovary (2) |
| chrX:54321213 |  |  | *WNK3* | p.T489M | * | Head_Neck (1) |
|  |  |  |  |  |  | Ovary (1) |
| chr17:7578461 |  |  | *TP53* | p.V157F | *** | Ovary (5) |
|  |  |  |  |  |  | Breast (1) |
| chr17:7579358 |  |  |  | p.R110L | ** (!) | Head_Neck (3) |
|  |  |  |  |  |  | Ovary (2) |
|  |  |  |  |  |  | Lung (1) |
| chr20:57767498 |  |  | *ZNF831* | p.T475M | unk | Melanoma (2) |
|  |  |  |  |  |  | Gastric (1) |
| chr3:134851754 |  |  | *EPHB1* | p.T387M | ** | Ovary (2) |
| chr6:105198338 |  |  | *HACE1* | p.V741F | ** | Head_Neck (1) |
|  |  |  |  | p.V741L |  | Ovary (1) |
| chr6:29455532 |  |  | *MAS1L* | p.V50I | * | Ovary (2) |
| chr7:93108747 |  |  | *CALCR* | p.V60I | * | Head_Neck (1) |
|  |  |  |  |  |  | Ovary (1) |
| chr8:120608097 |  |  | *ENPP2* | p.R373H | * | Colorectal (1) |
|  |  |  |  |  |  | Head_Neck (1) |
| chr12:111311719 | 47 | C**G**GA | *CCDC63* | p.R148Q | unk | Melanoma (2) |
| chr17:7574003 |  |  | *TP53* | p.R342X | *** | Ovary (3) |
|  |  |  |  |  |  | Head_Neck (2) |
| chr17:7578263 |  |  |  | p.R196X | ** (!) | Ovary (4) |
|  |  |  |  |  |  | Head_Neck (3) |
|  |  |  |  |  |  | Gastric (2) |
|  |  |  |  |  |  | Glioblastoma (1) |
|  |  |  |  |  |  | Myeloma (1) |
| chr3:52440373 |  |  | *BAP1* | p.R227C | ** | Ovary (2) |
| chr4:153247289 |  |  | *FBXW7* | p.R505G | ** | Head_Neck (2) |
|  |  |  |  | p.R505C | ** | Melanoma (1) |
|  |  |  |  |  |  | Ovary (1) |
| chr6:44220861 |  |  | *HSP90AB1* | p.R604Q | ** | Myeloma (1) |
|  |  |  |  | nr (1) |  | Prostate (1) |
| chr16:30455863 |  |  | *SEPHS2* | p.G396R | * | Breast (1) |
|  |  |  |  |  |  | Head_Neck (1) |
| chr17:7577538 |  |  | *TP53* | See above | See above | See above |
| chr5:13770873 |  |  | *DNAH5* | p.R3197Q | * | Ovary (2) |
| chr9:82319728 |  |  | *TLE4* | p.R214X | ** | Head_Neck (1) |
|  |  |  |  |  |  | Ovary (1) |
| chr2:10904525 | 38 | C**G**TG | *ATP6V1C2* | p.V118M | unk | Ovary (1) |
|  |  |  |  | p.V118L |  | Ovary (1) |
| chr20:57484421 |  |  | *GNAS* | p.R201H | *** | Colorectal (1) |
|  |  |  |  |  |  | Pancreatic (1) |
| chr3:10974900 |  |  | *SLC6A11* | p.V479M | unk | Melanoma (2) |
| chr3:183774067 |  |  | *HTR3C* | p.V128M | unk | Colorectal (1) |
|  |  |  |  |  |  | Ovary (1) |
| chr3:77666779 |  |  | *ROBO2* | p.V1137M | ** | Colorectal (1) |
|  |  |  |  |  |  | Gastric (1) |
| chr1:186919850 |  |  | *PLA2G4A* | p.H442Q | * | Breast (1) |
|  |  |  |  |  |  | Ovary (1) |
| chr17:7577120 |  |  | *TP53* | See above | See above | See above |
| chr2:106761805 |  |  | *UXS1* | p.V100L | unk | Ovary (2) |
| chr4:38798159 |  |  | *TLR1* | p.R764H | ** | Lung (2) |
| chr5:36035828 |  |  | *UGT3A2* | p.R515H | unk | Colorectal (1) |
|  |  |  |  |  |  | Ovary (1) |
|  |  |  |  |  |  |  |
| ***C. DGAN sequences most often mutated in rNS substitutions*** | | | | | | |
|  |  |  |  |  |  |  |
| chr1:3328165 | 20 | T**G**AT | *PRDM16* | p.M468I | ** | Lung (2) |
| chr12:6439046 |  |  | *TNFRSF1A* | p.Q319X | * | Head_Neck (1) |
|  |  |  |  |  |  | Lung (1) |
| chr12:66641637 |  |  | *IRAK3* | p.D493N | ** | Head_Neck (1) |
|  |  |  |  |  |  | Lung (1) |
| chr3:119624622 |  |  | *GSK3B* | p.Q265X | ** | Lung (2) |
| chr3:155282830 |  |  | *PLCH1* | p.Q303X | * | Melanoma (2) |
| chr4:94006418 |  |  | *GRID2* | p.D173Y | * | Head_Neck (1) |
|  |  |  |  |  |  | Ovary (1) |
| chr7:143141184 |  |  | *TAS2R60* | p.M213I | unk | Melanoma (2) |
| chr2:202149893 |  |  | *CASP8* | p.S445X | ** | Head_Neck (2) |
| chr7:88956757 |  |  | *ZNF804B* | p.Q117X | unk | Head_Neck (2) |
| chrX:83128420 |  |  | *CYLC1* | p.S235X | unk | Liver (1) |
|  |  |  |  |  |  | Ovary (1) |
| chr12:126138484 | 26 | G**G**AA | *TMEM132B* | p.G822E | unk | Melanoma (2) |
| chr17:7577559 |  |  | *TP53* | p.S241F | ** (!) | Ovary (4) |
|  |  |  |  |  |  | Breast (1) |
|  |  |  |  | p.S241Y | ** (!) | Ovary (1) |
| chr18:50278496 |  |  | *DCC* | p.G55E | ** | Melanoma (2) |
| chr7:110763912 |  |  | *LRRN3* | p.E362K | ** | Melanoma (2) |
| chr16:9984854 |  |  | *GRIN2A* | p.E371K | ** | Melanoma (2) |
| chr17:7577082 |  |  | *TP53* | p.E286K | ** (!) | Head_Neck (2) |
|  |  |  |  |  |  | Ovary (1) |
| chr17:7578257 |  |  | *TP53* | p.E198X | ** (!) | Ovary (3) |
| chr18:40854006 |  |  | *SYT4* | p.E130K | * | Melanoma (2) |
| chr20:57829185 |  |  | *ZNF831* | p.S1474F | unk | Melanoma (4) |
| chr14:30135364 | 20 | T**G**AA | *PRKD1* | p.H152Y | unk | Breast (1) |
|  |  |  |  |  |  | Colorectal (1) |
| chr20:57829451 |  |  | *ZNF831* | p.E1563K | unk | Melanoma (2) |
| chr3:148583291 |  |  | *CPA3* | p.E34X | * | Breast (1) |
|  |  |  |  | p.E34K |  | Melanoma (1) |
| chr3:178936082 |  |  | *PIK3CA* | p.E542K | *** | Breast (1) |
|  |  |  |  |  |  | Colorectal (1) |
| chr3:178952074 |  |  | *PIK3CA* | p.M1043I | *** | Breast (2) |
| chr3:189456461 |  |  | *TP63* | p.L74F | ** | Head_Neck (2) |
| chr4:40356060 |  |  | *CHRNA9* |  | * | Head_Neck (1) |
|  |  |  |  |  |  | Melanoma (1) |
| chr17:7579503 |  |  | *TP53* | p.E62K | ** (!) | Ovary (2) |
| chr17:7579521 |  |  | *TP53* |  |  | Colorectal (1) |
|  |  |  |  | p.E56X | ** (!) | Head_Neck (1) |
| chr18:40854357 |  |  | *SYT4* | p.E13K | ** | Melanoma (2) |
