# Supplementary material for: Guanine Holes Are Prominent Targets for Mutation in Cancer and Inherited Disease
Source: PLoS Genet. 2013 Sep 26;9(9):e1003816. doi: 10.1371/journal.pgen.1003816 (PMC3784513; doi:10.1371/journal.pgen.1003816)
Supplement: Table S10 — Individual patient samples harboring genes with ≥4 NS substitutions. Sequence context-dependency of patient samples which experienced ≥4 NS substitutions in the same gene. White background, SBSs at DGNN sequences; white on black background, SBSs at CGNN sequences; gray background, SBSs at NANN sequences; bold, SBSs at DGRN sequences. Fraction of NGNN sequences: 30/35 = 86%). (DOCX) [file pgen.1003816.s015.docx]

| Sample ID | Tumor type | Gene ID | Chrom | Hg19 | cDNA change | Protein change | NRNN seq |
| --- | --- | --- | --- | --- | --- | --- | --- |
| Lung_nsc | LUNG | MUC16 | chr19 | 9048312 | c.C33522A | p.A11106S | GGCA |
|  |  |  | chr19 | 9062563 | c.G25086T | p.P8294T | **GGAA** |
|  |  |  | chr19 | 9063004 | c.C24645A | p.G8147C | **TGGT** |
|  |  |  | chr19 | 9063990 | c.C23659A | p.R7818M | **AGGA** |
|  |  |  | chr19 | 9074613 | c.G13036T | p.S4277Y | **GGAG** |
| HN_62854 | HEAD_NECK | SYNE2 | chr14 | 64457763 | c.C2806T | p.S859L | **TGAT** |
|  |  |  | chr14 | 64514793 | c.G7527A | p.E2433K | **TGAG** |
|  |  |  | chr14 | 64537372 | c.G10671C | p.E3481Q | **AGAG** |
|  |  |  | chr14 | 64626137 | c.G16301C | p.E5357D | **AGAA** |
|  |  |  | chr14 | 64634238 | c.G17016A | p.E5596K | **TGAA** |
| HN_62741 | HEAD_NECK | COL11A1 | chr1 | 103352525 | c.G5050A | p.D1578N | **AGAT** |
|  |  |  | chr1 | 103387097 | c.C4039A | p.P1241T | **GGGC** |
|  |  |  | chr1 | 103388936 | c.G3964A | p.G1216S | **AGGC** |
|  |  |  | chr1 | 103427444 | c.C3500A | p.P1061H | **GGGA** |
|  |  |  | chr1 | 103431047 | c.C3266G | p.P983R | **TGGT** |
| HN22PT | HEAD_NECK | SYNE1 | chr6 | 152470709 | c.C24545T | p.A8111V | TGCA |
|  |  |  | chr6 | 152552651 | c.C20914T | p.Q6901X | **TGGT** |
|  |  |  | chr6 | 152671842 | c.G11644A | p.A3867T | AGCT |
|  |  |  | chr6 | 152763359 | c.G3859C | p.A1294P | AGCA |
| HX16T | LIVER | DKFZp667P0924 | chr2 | 96525750 | c.A2313T | p.H279L | CACT |
|  |  |  | chr2 | 96525755 | c.T2308A | p.D277E | TATC |
|  |  |  | chr2 | 96525768 | c.C2295T | p.T273I | TGTT |
|  |  |  | chr2 | 96525771 | c.T2292G | p.L272X | TAAT |
| HN_62854 | HEAD_NECK | DNAH9 | chr17 | 11523087 | c.C1407G | p.H447D | TGCA |
|  |  |  | chr17 | 11725299 | c.G8838A | p.E2924K | **TGAA** |
|  |  |  | chr17 | 11757569 | c.G9825C | p.E3253Q | **TGAG** |
|  |  |  | chr17 | 11757695 | c.G9951A | p.D3295N | **GGAC** |
| HN_62426 | HEAD_NECK | MED1 | chr17 | 37563898 | c.C4788T | p.H1526Y | **TGAG** |
|  |  |  | chr17 | 37564353 | c.C4333T | p.S1374F | **AGAA** |
|  |  |  | chr17 | 37564668 | c.C4018T | p.S1269F | **AGAT** |
|  |  |  | chr17 | 37565005 | c.C3681G | p.P1157A | **GGAG** |
| HN_62897 | HEAD_NECK | USH2A | chr1 | 216138740 | c.G7426T | p.V2347L | CGTG |
|  |  |  | chr1 | 216251606 | c.G5784C | p.K1799N | AGTG |
|  |  |  | chr1 | 216424419 | c.A2380T | p.K665X | TAAG |
|  |  |  | chr1 | 216495281 | c.A1975T | p.T530S | AACA |

**Table S10.** *Individual patient samples harboring genes with ≥4 NS substitutions*
