# Supplementary material for: Guanine Holes Are Prominent Targets for Mutation in Cancer and Inherited Disease
Source: PLoS Genet. 2013 Sep 26;9(9):e1003816. doi: 10.1371/journal.pgen.1003816 (PMC3784513; doi:10.1371/journal.pgen.1003816)
Supplement: Text S1 — Supporting information. The text contains information on the distribution of NGNN sequences in the mappability files and in Segmental Duplications, a description of mutational mechanisms shared by similar cancer types and individual samples, the analysis of recurrent non-synonymous substitutions, and exemplary scripts for obtaining the fractions f i of mutated NGNN sequences. (DOCX) [file pgen.1003816.s016.docx]

**Guanine Holes Are Prominent Targets for Mutation in Cancer and Inherited Disease**

Albino Bacolla, Nuri A. Temiz, Ming Yi, Joseph Ivanic, Regina Z. Cer, Duncan E. Donohue, Edward V. Ball, Uma S. Mudunuri, Guliang Wang, Aklank Jain, Natalia Volfovsky, Brian T. Luke, Robert M. Stephens, David N. Cooper, Jack R. Collins, and Karen M. Vasquez

**TEXT S1**

**Mappability and the Enrichment of CGNN Sequences Within Segmental Duplications**

The presence of highly homologous regions, *i.e.* Segmental Duplications (SD), repetitive DNA elements and simple repeats, limit the total number of observable mutations to those unique portions of the human genome that may be efficiently mapped by next-generation sequencing. Thus, we first compared the relative abundances of NGNN sequences in the whole genome (T_hg19) with those obtained in SD, repetitive elements plus simple repeats (RM) and the “mappable” genome, as obtained from two methods (*i.e.* Duke35 and CGR50; see Materials and Methods; Table S1, Panel *A*). Similar comparisons were also performed for the exonic NGNN sequences between genome-wide RefSeq exons (T_exons), exons targeted by the SureSelect Human Exon platform (AgilentV2), and the mappable exons from CRG50 (CRG50_exons; see Materials and Methods; Table S1, Panel *B*).

Duke35 and CRG50 revealed a significant deficiency (-6 and -10%, respectively) in CGNN sequences (P-values 7.20 x 10^-5^ and 1.36 x 10^-6^, respectively, *t*-test; Table S2), compared to the whole genome, as a result of excess (~20%, P-value 1.03 x 10^-14^) CGNN motifs in SD. 3G motifs, *i.e.* 75 – 100% C+G-rich NGNN sequences, were also underrepresented (-2.6 and -3.3%) in Duke35 and CRG50 (P-values 0.02) as a result of their excess (5.9%) in SD (P-value 2.34 x 10^-4^). By contrast, no differences in the relative representation of non-CGNN sequences were noted between motifs that contained a purine and motifs that contained a pyrimidine at the third position (*i.e.* DGRN *vs*. DGYN, D = A/G/T). For the NGNN sequences in exons, CGNN motifs were found to be significantly overrepresented (0.8%) in CRG50_exons relative to all RefSeq exons (T_exons) (P-value 1.77 x 10^-3^), a difference that was likely masked in AgilentV2 by a high standard deviation. In summary, whereas significant differences existed in the relative representations of CGNN sequences between the whole genome, or exome, and their mappable counterparts, no differences were noted between DGRN and DGYN motifs.

**Similar Cancer Types and Individual Samples Share Mutational Patterns**

To verify the conclusion that *f*(NGNN) varied between classes of NGNN sequences by cancer type, we addressed whether *f*(NGNN) varied between cancer types more than they did between individual samples within cancer datasets. To this end, we compared *f*(NGNN) for the tumor samples comprising two contrasting cancer datasets, Pancreatic_ca, a large GWS dataset that included 5 samples and displayed ~5-fold higher mutation fractions at CGNN than at DGNN (P-values 1.6 x 10^-21^ – 2.6 x 10^-26^, Table S4, Panel *B*) but no differences between DGRN and DGYN, and Melanoma_ews, a small EWS dataset comprising 14 samples, and which displayed 5-fold higher mutation fractions at DGRN than at DGYN (P-values 1.2 x 10^-4^ – 8.8 x 10^-5^, Table S4, Panel *C*) and modest (P-values 0.018 – 0.023) differences between CGNN and DGNN (Table 1 and Table S4, Panels *B – D*).

Each one of the 5 Pancreatic_ca tumor samples displayed 3 – 10-fold higher mutation fractions at CGNN than at DGNN (P-values from t-tests in the range 5.4 x 10^-10^ to 1.2 x 10^-31^, whereas only one (PCSI_0022_Pa_P_01) exhibited a slight (1.3-fold) increase in mutation fractions at CGNN relative to DGNN (P-values 0.01 – 0.02) (Table S4, Panel *B*). These data were consistent irrespective of the mapping method used. By contrast, with the exception of sample 35T, whose number of SBSs was the smallest (17 total), all Melanoma_ews samples displayed significantly more mutations at DGRN than at DGYN (mean P(α)_0.05_ > 0.99 as assessed from *z*-tests). These results were strikingly consistent given that the coefficient of variation for P(α)_0.05_ was ≤5.8% for all mapping methods used (Table S4, Panel *D*). With respect to the differences between CGNN and DGNN, samples were evenly split, half showing *f*(CGNN)>*f*(DGNN) while the other half displaying no difference. Further statistical comparisons between the relative fractions of mutated CGNN and DGNN for the 14 Melanoma_ews samples did indicate significantly higher mutations at CGNN sequences (mean ± SD: CGNN, 0.65 ± 0.06; DGNN, 0.35 ± 0.06; P-value <0.001, Holm-Sidak test after Shapiro-Wilk normality test), while confirming more frequent mutations at DGRN than at DGYN sequences (median: 0.53 for DGRN and 0.11 for DGYN; P-value <0.001, Kruskal-Wallis one way analysis of variance on ranks) (Figure S1, Panel *A*).

The large number of SBSs for the 5 Pancreatic_au samples enabled an assessment of their position relative to all other cancer and germline datasets by means of agglomerative hierarchical clustering, based on the Manhattan distances computed from all 1600 *f*(NGNN) values (64 x 25 datasets). Three notable features were revealed: first, all 5 pancreatic cancer samples clustered together on the lowest branch, along with the Pancreatic_ca, Pancreatic_au and Prostate datasets; second, the two melanoma datasets split from all other cancer datasets at the root of the tree; and third, with the exception of Lung_sc, an established cell line, the two pancreatic (one GWS the other EWS), the two melanoma (one GWS the other EWS) and the two liver (both GWS) datasets clustered next to each other.

In summary, we conclude that the differences observed in mutational patterns among classes of NGNN sequences (i.e. CGNN *vs*. DGNN and DGRN *vs*. DGYN) are shaped primarily by underlying biological processes, and to a lesser extent by variability in next-generation sequencing methodologies and/or individual sample variation.

**Recurrent Non-Synonymous Substitutions**

A total of 14,172 genes with NS substitutions were retrieved with variable fractional representation in the tumor tissues (Table S8). Since large dataset analyses are necessary for the identification of driver mutations [[104](#_ENREF_1)], we retrieved the genes with the most NS substitutions in the combined datasets. *TP53* displayed the highest number of mutations as expected, with 480, followed by *TTN* (titin) and *MUC16* (mucin-16) with 130 and 93 occurrences, respectively. Although titin and mucin-16 are the two largest known proteins encoded by the human genome and hence their genes represent large mutational targets, their role in cancer has not been firmly established; for this reason, we normalized the numbers of NS substitutions by the number of amino acids (*H*). For the 29 genes with ≥24 SBSs, *TP53*, *KRAS* and *PIK3CA* were the highest-ranking entries, whereas *TTN* and *MUC16* ranked towards the bottom, suggesting that SBSs in these genes were not drivers of tumorigenesis (Figure S3, Panel *A*). The high *H* values for the cancer genes, *TP53*, *KRAS* and *PIK3CA*, were largely attributable to recurrent mutations, *i.e.*, SBSs that occurred at the same genomic coordinate in two or more patient samples, supporting the view that recurrent NS substitutions may be enriched in *bona fide* driver mutations.

There were 35,480 NS substitutions in the combined cancer datasets, 78% of which altered G•C bps; G→A transitions represented the most common type of substitution, accounting for ~50% of all SBSs (Figure S3, Panel *C*). A total of 972/35,480 NS substitutions (2.7%) recurred (2-41 instances) at 302 genomic coordinates, 74% of which (224 total) occurred at G•C bp. Thus, both the NS and recurrent NS sets of SBSs shared with the EWS datasets (Figure 1, Panel *A*) a preponderance of mutations at G•C bps. Of the 150 genes recurrently mutated, those with ≥4 recurrent NS mutations (26 total, Figure S3, Panel *B*) displayed higher *H* values (median = 1.75) than the genes with the highest numbers of NS substitutions (median = 0.7) (Figure S3, Panel *A*); the recurrent NS mutations also tended to affect lower molecular weight proteins with predicted roles in cancer.

**Exemplary Scripts for Obtaining the Fractions, *f*_i_, of Mutated NGNN**

**Step 1** – Determine the number of mutated NGNN. This script is specific for the study by Lee et al. Nature 465, 473, 2010. In all scripts the paths to files need to be reset.

Usage: ./leeOnly.script lee_Original.txt

#! /bin/bash

# leeOnly.script -- Calculate the number of mutated NGNN from S. Table 5 of Lee et al., Nature 465, 473, 2010

# commands specific to this table (original data are in hg18)

cp $1 tmp1.txt

egrep -w "snp" tmp1.txt |

cut -f2,4 |

sed '

s/chrXnonPAR/chrX/g

s/PAR2/chrX/g

s/PAR1/chrX/g

s/chrYnonPAR/chrY/g' |

awk 'BEGIN { OFS = "\t" }

{ print $1,$2-2,$2+2 }' |

sed '

s/chrX/chr23/g

s/chrY/chr24/g

/random/d' |

sort -n -k1.4 -k2 |

sed '

s/chr23/chrX/g

s/chr24/chrY/g' > tmp2.txt

# script common to datasets with coordinates in hg18

/tetraCountCommonHg18.script

#! /bin/bash

# tetraCountCommonHg18.script -- commands common to all tables from file tmp2.txt containing "chr" "start" "stop"

/corral-repl/utexas/EBI_Cancer_Genome/scripts/liftOver tmp2.txt /corral-repl/utexas/EBI_Cancer_Genome/scripts/hg18ToHg19.over.chain tmp3.txt unmapped.txt

awk '{ print $1":"$2-1"-"$3 }' tmp3.txt > pos.txt

/corral-repl/utexas/EBI_Cancer_Genome/scripts/twoBitToFa -seqList=pos.txt /corral-repl/utexas/EBI_Cancer_Genome/ucsc_original/hg19.2bit seq.fa

egrep -v ">" seq.fa | tr '[A-Z]' '[a-z]' > tmp4.txt

cut -c3 tmp4.txt > tmp5.txt

a=`grep "[acgt]" tmp5.txt | wc -l`

b=`grep "[cg]" tmp5.txt | wc -l`

awk 'BEGIN { OFS "\t" } { print $1 "_" $2 + 2 }' tmp3.txt > tmp6.txt

paste tmp6.txt tmp5.txt | tr '\t' '_' > position.txt

cat tmp4.txt |

awk -v to=$a -v cg=$b 'BEGIN { OFS = "\t" }

/.agaa/ { count["agaa"]++ }

/.agac/ { count["agac"]++ }

/.agag/ { count["agag"]++ }

/.agat/ { count["agat"]++ }

/.agca/ { count["agca"]++ }

/.agcc/ { count["agcc"]++ }

/.agcg/ { count["agcg"]++ }

/.agct/ { count["agct"]++ }

/.agga/ { count["agga"]++ }

/.aggc/ { count["aggc"]++ }

/.aggg/ { count["aggg"]++ }

/.aggt/ { count["aggt"]++ }

/.agta/ { count["agta"]++ }

/.agtc/ { count["agtc"]++ }

/.agtg/ { count["agtg"]++ }

/.agtt/ { count["agtt"]++ }

/.cgaa/ { count["cgaa"]++ }

/.cgac/ { count["cgac"]++ }

/.cgag/ { count["cgag"]++ }

/.cgat/ { count["cgat"]++ }

/.cgca/ { count["cgca"]++ }

/.cgcc/ { count["cgcc"]++ }

/.cgcg/ { count["cgcg"]++ }

/.cgct/ { count["cgct"]++ }

/.cgga/ { count["cgga"]++ }

/.cggc/ { count["cggc"]++ }

/.cggg/ { count["cggg"]++ }

/.cggt/ { count["cggt"]++ }

/.cgta/ { count["cgta"]++ }

/.cgtc/ { count["cgtc"]++ }

/.cgtg/ { count["cgtg"]++ }

/.cgtt/ { count["cgtt"]++ }

/.ggaa/ { count["ggaa"]++ }

/.ggac/ { count["ggac"]++ }

/.ggag/ { count["ggag"]++ }

/.ggat/ { count["ggat"]++ }

/.ggca/ { count["ggca"]++ }

/.ggcc/ { count["ggcc"]++ }

/.ggcg/ { count["ggcg"]++ }

/.ggct/ { count["ggct"]++ }

/.ggga/ { count["ggga"]++ }

/.gggc/ { count["gggc"]++ }

/.gggg/ { count["gggg"]++ }

/.gggt/ { count["gggt"]++ }

/.ggta/ { count["ggta"]++ }

/.ggtc/ { count["ggtc"]++ }

/.ggtg/ { count["ggtg"]++ }

/.ggtt/ { count["ggtt"]++ }

/.tgaa/ { count["tgaa"]++ }

/.tgac/ { count["tgac"]++ }

/.tgag/ { count["tgag"]++ }

/.tgat/ { count["tgat"]++ }

/.tgca/ { count["tgca"]++ }

/.tgcc/ { count["tgcc"]++ }

/.tgcg/ { count["tgcg"]++ }

/.tgct/ { count["tgct"]++ }

/.tgga/ { count["tgga"]++ }

/.tggc/ { count["tggc"]++ }

/.tggg/ { count["tggg"]++ }

/.tggt/ { count["tggt"]++ }

/.tgta/ { count["tgta"]++ }

/.tgtc/ { count["tgtc"]++ }

/.tgtg/ { count["tgtg"]++ }

/.tgtt/ { count["tgtt"]++ }

/ttct./ { count["ttct"]++ }

/gtct./ { count["gtct"]++ }

/ctct./ { count["ctct"]++ }

/atct./ { count["atct"]++ }

/tgct./ { count["tgct1"]++ }

/ggct./ { count["ggct1"]++ }

/cgct./ { count["cgct1"]++ }

/agct./ { count["agct1"]++ }

/tcct./ { count["tcct"]++ }

/gcct./ { count["gcct"]++ }

/ccct./ { count["ccct"]++ }

/acct./ { count["acct"]++ }

/tact./ { count["tact"]++ }

/gact./ { count["gact"]++ }

/cact./ { count["cact"]++ }

/aact./ { count["aact"]++ }

/ttcg./ { count["ttcg"]++ }

/gtcg./ { count["gtcg"]++ }

/ctcg./ { count["ctcg"]++ }

/atcg./ { count["atcg"]++ }

/tgcg./ { count["tgcg1"]++ }

/ggcg./ { count["ggcg1"]++ }

/cgcg./ { count["cgcg1"]++ }

/agcg./ { count["agcg1"]++ }

/tccg./ { count["tccg"]++ }

/gccg./ { count["gccg"]++ }

/cccg./ { count["cccg"]++ }

/accg./ { count["accg"]++ }

/tacg./ { count["tacg"]++ }

/gacg./ { count["gacg"]++ }

/cacg./ { count["cacg"]++ }

/aacg./ { count["aacg"]++ }

/ttcc./ { count["ttcc"]++ }

/gtcc./ { count["gtcc"]++ }

/ctcc./ { count["ctcc"]++ }

/atcc./ { count["atcc"]++ }

/tgcc./ { count["tgcc1"]++ }

/ggcc./ { count["ggcc1"]++ }

/cgcc./ { count["cgcc1"]++ }

/agcc./ { count["agcc1"]++ }

/tccc./ { count["tccc"]++ }

/gccc./ { count["gccc"]++ }

/cccc./ { count["cccc"]++ }

/accc./ { count["accc"]++ }

/tacc./ { count["tacc"]++ }

/gacc./ { count["gacc"]++ }

/cacc./ { count["cacc"]++ }

/aacc./ { count["aacc"]++ }

/ttca./ { count["ttca"]++ }

/gtca./ { count["gtca"]++ }

/ctca./ { count["ctca"]++ }

/atca./ { count["atca"]++ }

/tgca./ { count["tgca1"]++ }

/ggca./ { count["ggca1"]++ }

/cgca./ { count["cgca1"]++ }

/agca./ { count["agca1"]++ }

/tcca./ { count["tcca"]++ }

/gcca./ { count["gcca"]++ }

/ccca./ { count["ccca"]++ }

/acca./ { count["acca"]++ }

/taca./ { count["taca"]++ }

/gaca./ { count["gaca"]++ }

/caca./ { count["caca"]++ }

/aaca./ { count["aaca"]++ }

END { print "tetra", "count"

print "agaa", count["agaa"] + count["ttct"]

print "agac", count["agac"] + count["gtct"]

print "agag", count["agag"] + count["ctct"]

print "agat", count["agat"] + count["atct"]

print "agca", count["agca"] + count["tgct1"]

print "agcc", count["agcc"] + count["ggct1"]

print "agcg", count["agcg"] + count["cgct1"]

print "agct", count["agct"] + count["agct1"]

print "agga", count["agga"] + count["tcct"]

print "aggc", count["aggc"] + count["gcct"]

print "aggg", count["aggg"] + count["ccct"]

print "aggt", count["aggt"] + count["acct"]

print "agta", count["agta"] + count["tact"]

print "agtc", count["agtc"] + count["gact"]

print "agtg", count["agtg"] + count["cact"]

print "agtt", count["agtt"] + count["aact"]

print "cgaa", count["cgaa"] + count["ttcg"]

print "cgac", count["cgac"] + count["gtcg"]

print "cgag", count["cgag"] + count["ctcg"]

print "cgat", count["cgat"] + count["atcg"]

print "cgca", count["cgca"] + count["tgcg1"]

print "cgcc", count["cgcc"] + count["ggcg1"]

print "cgcg", count["cgcg"] + count["cgcg1"]

print "cgct", count["cgct"] + count["agcg1"]

print "cgga", count["cgga"] + count["tccg"]

print "cggc", count["cggc"] + count["gccg"]

print "cggg", count["cggg"] + count["cccg"]

print "cggt", count["cggt"] + count["accg"]

print "cgta", count["cgta"] + count["tacg"]

print "cgtc", count["cgtc"] + count["gacg"]

print "cgtg", count["cgtg"] + count["cacg"]

print "cgtt", count["cgtt"] + count["aacg"]

print "ggaa", count["ggaa"] + count["ttcc"]

print "ggac", count["ggac"] + count["gtcc"]

print "ggag", count["ggag"] + count["ctcc"]

print "ggat", count["ggat"] + count["atcc"]

print "ggca", count["ggca"] + count["tgcc1"]

print "ggcc", count["ggcc"] + count["ggcc1"]

print "ggcg", count["ggcg"] + count["cgcc1"]

print "ggct", count["ggct"] + count["agcc1"]

print "ggga", count["ggga"] + count["tccc"]

print "gggc", count["gggc"] + count["gccc"]

print "gggg", count["gggg"] + count["cccc"]

print "gggt", count["gggt"] + count["accc"]

print "ggta", count["ggta"] + count["tacc"]

print "ggtc", count["ggtc"] + count["gacc"]

print "ggtg", count["ggtg"] + count["cacc"]

print "ggtt", count["ggtt"] + count["aacc"]

print "tgaa", count["tgaa"] + count["ttca"]

print "tgac", count["tgac"] + count["gtca"]

print "tgag", count["tgag"] + count["ctca"]

print "tgat", count["tgat"] + count["atca"]

print "tgca", count["tgca"] + count["tgca1"]

print "tgcc", count["tgcc"] + count["ggca1"]

print "tgcg", count["tgcg"] + count["cgca1"]

print "tgct", count["tgct"] + count["agca1"]

print "tgga", count["tgga"] + count["tcca"]

print "tggc", count["tggc"] + count["gcca"]

print "tggg", count["tggg"] + count["ccca"]

print "tggt", count["tggt"] + count["acca"]

print "tgta", count["tgta"] + count["taca"]

print "tgtc", count["tgtc"] + count["gaca"]

print "tgtg", count["tgtg"] + count["caca"]

print "tgtt", count["tgtt"] + count["aaca"]

print "total ", to

print "ratio ", cg/to

}' > tetraCount.txt

rm tmp[1-6].txt pos.txt seq.fa

#end

**Step 2** – Determine the NGNN counts for the mappable genome. The script exemplifies “Duke35”. Note: tetcount, tetcount.f and 4mer_patt.txt are files of a custom FORTRAN script to fetch tetranucleotide repeats.

#! /bin/bash

# ngnnInDuke35.script -- get tetracount from file wgEncodeDukeMapabilityUniqueness35bp.bedGraph to use as mappable genome

# select regions with mappability = 1

awk '$4 == 1' wgEncodeDukeMapabilityUniqueness35bp.bedGraph |

sed '

/chrM/d

s/chr17 0 884 1/chr17 2 884 1/g' > tmp0.txt

awk '{ print $1 ":" $2 - 2 "-" $3 + 2 }' tmp0.txt > tmp1.txt

/corral-repl/utexas/EBI_Cancer_Genome/scripts/twoBitToFa -seqList=tmp1.txt /corral-repl/utexas/EBI_Cancer_Genome/ucsc_original/hg19.2bit tmp1.fa

cp /corral-repl/utexas/EBI_Cancer_Genome/brian/tetcount .

cp /corral-repl/utexas/EBI_Cancer_Genome/brian/tetcount.f .

cp /corral-repl/utexas/EBI_Cancer_Genome/brian/4mer_patt.txt .

./tetcount tmp1.fa tmp2.txt

egrep -w ".G.." tmp2.txt > tmp3.txt

mv tmp2.txt tetInDuke35.txt

mv tmp3.txt ngnnInDuke35.txt

rm tmp0.txt tmp1.txt tmp7.txt tmp1.fa tetcount tetcount.f 4mer_patt.txt

**Step 3** – Determine the fraction of mutated NGNN#! /bin/bash

# dukeNgnnFractions.script -- calculate fractions of mutated NGNN in GWS cancer datasets using Duke35 mappable genome

a=/corral-repl/utexas/EBI_Cancer_Genome

b=/corral-repl/utexas/EBI_Cancer_Genome

# 0) -- get full list of NGNN sequences

cut -f1,3 $a/ngnnInDuke35.txt | grep -w ".G.." > tmp1.txt

cut -f2,3 $a/ngnnInDuke35.txt | grep -w ".G.." >> tmp1.txt

sort -d tmp1.txt |

awk 'BEGIN { OFS = "\t" }

{

if ($1 ~ /^AGCT$/ || $1 ~ /^CGCG$/ || $1 ~ /^GGCC$/ || $1 ~ /^TGCA$/)

print $1, $2 = 2 * $2

else

print $1, $2

}' | sort -u > ngnnDukeList.txt

rm tmp1.txt

# 4) -- Lee - Lung_nsc

egrep -w "[acgt][acgt]+" $b/lee/tetraCount.txt |

paste - ngnnDukeList.txt |

awk 'BEGIN { OFS = "\t" }

{ print $3, $2 = $2/$4 }' > dukeShortFractionsLung_nsc.txt

cp dukeShortFractionsLung_nsc.txt tmp1.txt

./sortNgnnFractions.script > dukeLongFractionsLung_nsc.txt

rm tmp1.txt

#! /bin/bash

# sortNgnnFractions.script -- divide tetra fractions into classes for statistical analyses

cat tmp1.txt |

awk 'BEGIN { OFS = "\t" }

{

if ($1 ~ /^CG..$/)

print "1", "CGNN", $0

if ($1 !~ /^CG..$/)

print "2", "DGNN", $0

if ($1 ~ /[GAT]G[AG][ACGT]/ && $1 !~ /^CG..$/)

print "3", "DGRN", $0

if ($1 ~ /[GAT]G[CT][ACGT]/ && $1 !~ /^CG..$/)

print "4", "DGYN", $0

}' |

sort –u

**Disclaimer**

The content of this publication does not necessarily reflect the views or policies of the Department of Health and Human Services, nor does mention of trade names, commercial products, or organizations imply endorsement by the U.S. government.

**Supplemental Reference**

104. Vandin F, Upfal E, Raphael BJ (2012) *De novo* discovery of mutated driver pathways in cancer. Genome Res 22: 375-385.
